# A driven Kerr oscillator with two-fold degeneracies for qubit protection

**DOI:** 10.1073/pnas.2311241121

**Published:** 2024-06-05

**Authors:** Jayameenakshi Venkatraman, Rodrigo G. Cortiñas, Nicholas E. Frattini, Xu Xiao, Michel H. Devoret

**Affiliations:** ^a^Department of Applied Physics, Yale University, New Haven, CT 06520; ^b^Department of Physics, Yale University, New Haven, CT 06520

**Keywords:** quantum computing, quantum information, symmetries, floquet systems, tunneling

## Abstract

We present the experimental finding of multiple simultaneous two-fold degeneracies in the spectrum of a Kerr oscillator subjected to a squeezing drive. This squeezing drive resulting from a three-wave mixing process, in combination with the Kerr interaction, creates an effective static two-well potential in the phase space rotating at half the frequency of the sinusoidal drive generating the squeezing. Remarkably, these degeneracies can be turned on-and-off on demand, as well as their number by simply adjusting the frequency of the squeezing drive. We find that when the detuning Δ between the frequency of the oscillator and the second subharmonic of the drive equals an even multiple of the Kerr coefficient *K*, Δ/K=2m, the oscillator displays m+1 exact, parity-protected, spectral degeneracies, insensitive to the drive amplitude. These degeneracies can be explained by the unusual destructive interference of tunnel paths in the classically forbidden region of the double well static effective potential that models our experiment. Exploiting this interference, we measure a peaked enhancement of the incoherent well-switching lifetime, thus creating a protected cat qubit in the ground state manifold of our oscillator. Our results illustrate the relationship between degeneracies and noise protection in a driven quantum system.

## Significance

In the quantum world, it seems there is always a path to escape from a trap, thanks to quantum tunneling. However, counterintuitively, if a second path is also available, the two paths can be arranged so that they destructively interfere with each other, thus blocking the escape. This interference is also a quantum mechanical feature and enters here to null out the freedom provided by quantum tunneling. By controlling the phase difference between the two paths, one can control tunneling in a way that is completely independent of the barrier height, a phenomenon with applications in quantum computation, in molecular and nuclear physics. Our experiment has demonstrated this interference effect in a controlled tunneling double-well system.

Energy level degeneracies and their connection to symmetries play a pivotal role in physics. For instance, atoms like the hydrogen atom present energy level degeneracies connected to spherical symmetry of the attractive potential of the nucleus. In quantum information, such symmetry favors the emergence of a noise-protected manifold of two states, if the symmetry of the states prevents the environment to distinguish between them. For example, topological quantum systems exhibit global symmetries that result in degenerate ground states with inherent protection against local noise (1). The pursuit of noise protection in qubits has led to the proposal and investigation of complex novel circuits, such as the 0−π qubit (2) and the cos2φ qubit (3, 4), whose near-degenerate qubit states are endowed with inherent resilience to decay and dephasing. However, the realization of such protected qubits often demands finely tuned circuit parameters that tend to drift, like the flux through a superconducting loop (5, 6).

Encoding a protected qubit in a driven system provides a means of tuning the protection in situ by adjusting the drive parameters. A driven Kerr oscillator readily provides such a degenerate manifold of ground states that are stabilized under dissipation. This can be shown (7) by taking a Kerr nonlinear oscillator, subject to a two-photon squeezing drive and finding that its Hamiltonian in the rotating frame of the oscillator $\hat{H}/\hbar =-K{\hat{a}}^{†2}{\hat{a}}^{2}+\epsilon_{2}\left({\hat{a}}^{†2}+{\hat{a}}^{2}\right)$ can be written into a factorizable form as $\hat{H}/\hbar =-K\left({\hat{a}}^{†2}-\epsilon_{2}/K\right)\left({\hat{a}}^{2}-\epsilon_{2}/K\right)+\epsilon_{2}^{2}/K$, where $\hat{a}$ is the annihilation operator, *K* is the Kerr coefficient, and $\epsilon_{2}$ the strength of the squeezing interaction. The coherent states $|\alpha =\pm \sqrt{\epsilon_{2}/K}\rangle$, which are the eigenstates of the annihilation operator associated with photon-loss, are degenerate eigenstates of the Hamiltonian. This property was key for the proposal of the Kerr-cat qubit (7, 8), which was realized experimentally (9, 10).

It is worthwhile to ask whether the protection associated with the Hamiltonian factorization is a special feature or just one instance of a more general phenomenon. For instance, how would the introduction of a simple parameter such as a detuning,

[1] $$\hat{H}/\hbar =\Delta {\hat{a}}^{†}\hat{a}-K{\hat{a}}^{†2}{\hat{a}}^{2}+\epsilon_{2}\left({\hat{a}}^{†2}+{\hat{a}}^{2}\right)$$

affect the degeneracy. At first glance, introducing Δ≠0 seems to be a terrible idea since the beautiful factorization, leading to the exponentially small sensitivity of its spectrum to drive frequency fluctuations, is broken.

However, in this paper, we experimentally demonstrate that introducing detuning actually improves the attractiveness of the Kerr-cat qubit. We identify a counterintuitive phenomenon: a family of tunable parity-protected degeneracies that occur not only in the ground state manifold, but also in the excited state manifolds of our system. Specifically, we observe that when the harmonic term controlled by the parameter Δ in Eq. **1** equals an even multiple of the Kerr coefficient *K*, Δ/K=2m, the oscillator displays m+1 exact, parity-protected, spectral degeneracies that are insensitive to the amplitude of the squeezing drive $\epsilon_{2}$. Remarkably, these degeneracies correspond to the complete suppression of tunneling, not only for the ground state (11), but also for excited states (12) below the finite height barrier in the double-well potential. Our experiment realizes an elementary quantum system previously investigated only theoretically (11–15), and illustrates a means of fighting decoherence. Specifically, we show that the quantum states at the bottom of the double well form a qubit manifold with interwell transition lifetime that peaks when varying the drive frequency, while remaining addressable. This stems from the qubit manifold being not only protected from photon losses, but also from photon gain. This type of driven qubit could be useful as an ancilla for fault-tolerant syndrome measurement in quantum error correction (15–17).

## Experimental Implementation.

**Fig. 1.**
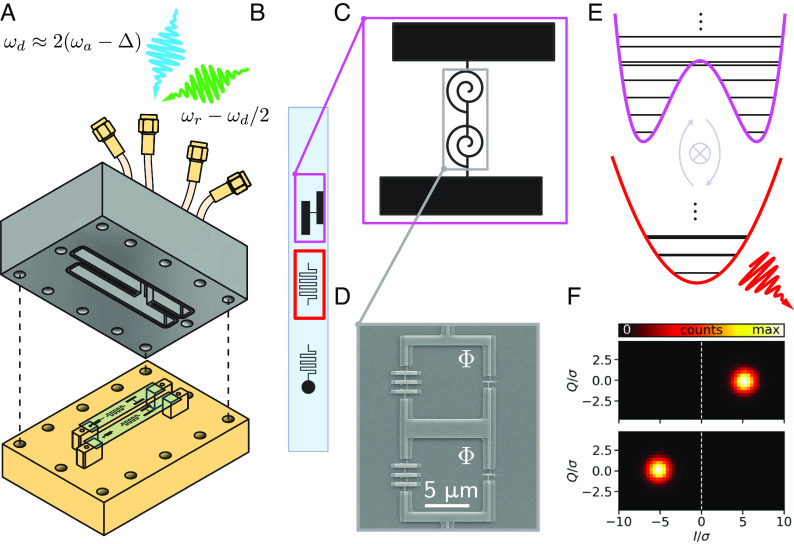
Experimental device overview. (*A*) Superconducting circuit package that houses two chips each containing a SNAIL-transmon circuit oscillator, readout mode, and Purcell filter. The blue and green arrows represent the microwave drives for squeezing and readout respectively. (*B*) Schematic zooming into a single chip containing lithographically embedded structures corresponding to a SNAIL transmon (pink rectangle), readout resonator (red rectangle), and a Purcell filter. (*C*) Schematic zooming in further to the SNAIL transmon which houses the bosonic mode in Eq. **1**. (*D*) Scanning electron micrograph of the array of two SNAILs. (*E*) Effective potential at p=0 associated with the Kerr-parametric oscillator and potential of the readout resonator, with the arrows representing the engineered beam splitter operation to transfer excitations from the SNAIL transmon to the readout resonator. (*F*) Readout histogram of the resonator output field demonstrating high-fidelity discrimination of the localized well-states. Package and device are reproduced from ref. 10 for reader convenience.

We implement the Hamiltonian Eq. **1** in a microwave-driven superconducting circuit that we now introduce. This setup was first introduced in ref. 9, with package first introduced in ref. 10 and summarized here for the sake of completeness. Fig. 1*A* shows a schematic of the superconducting circuit package. The package consists of two rectangular waveguide cavities (18) and the package an Aluminum top part and a Copper bottom. This arrangement results from a compromise between cavity quality factor and control of magnetic flux bias of the superconducting circuit. Each rectangle on the bottom-half of the package schematic represents a Sapphire chip clamped to two copper posts and shows three electron-beam-lithographically patterned structures corresponding to the three modes of interest for each cavity: a Superconducting Nonlinear Asymmetric Inductive eLement (SNAIL)-transmon (9, 19), a readout resonator, and a Purcell filter. Since only one rectangular cavity and its modes of interest are individually addressed in this work, we neglect the presence of the other “spectator” cavity and the second superconducting chip within. Fig. 1*B* shows a zoom-in to the chip of used in this work.

Fig. 1*C* shows a zoom-in schematic of the SNAIL-transmon array, and Fig. 1*D* shows a scanning electron micrograph further zooming in on the Josephson junctions. A SNAIL consists of a Josephson junction shunted by an array of larger Josephson junctions (9). While an ordinary Josephson junction is the circuit analog of a rigid pendulum, which has a cosine potential with the phase across the junction and the charge through the islands analogous to the angular position and angular momentum of the pendulum (see *SI Appendix*, Table 1 for expansion on the analogy), in the present case, the loop introduced by the shunting junctions, when threaded with an external magnetic field, provides an asymmetry of the potential. Therefore, the SNAIL-transmon should be understood as the circuit implementation of an asymmetric pendulum. Importantly, the SNAIL-transmon functions as a weakly nonlinear oscillator with three, and four-wave mixing which can be seen by expanding the Taylor series potential of an asymmetric cosine potential. We model the nonlinear oscillator associated with the SNAIL Hamiltonian as

[2] $$\begin{array}{c} \begin{array}{c} \hat{H}/\hbar =\omega_{o}{\hat{a}}^{†}\hat{a}+\frac{g_{3}}{3}{\left(\hat{a}+{\hat{a}}^{†}\right)}^{3}+\frac{g_{4}}{4}{\left(\hat{a}+{\hat{a}}^{†}\right)}^{4}. \end{array} \end{array}$$

In Eq. **2**, the classical small oscillation frequency of the oscillator is $\omega_{o}$, and the three-wave and four-wave mixing nonlinear coefficients are characterized by $g_{3},g_{4}$. The parameters, $\omega_{o}$, $g_{3}$, and $g_{4}$ can be tuned in situ by varying the external magnetic field.

The squeezing operation is facilitated by applying a microwave drive to a weakly coupled pin shown in Fig. 1*A*. In the presence of the drive, Eq. **2** is modified as

[3] $$\begin{array}{c} \begin{array}{cc} \hat{H}\left(t\right)/\hbar = & \omega_{o}{\hat{a}}^{†}\hat{a}+\frac{g_{3}}{3}{\left(\hat{a}+{\hat{a}}^{†}\right)}^{3}+\frac{g_{4}}{4}{\left(\hat{a}+{\hat{a}}^{†}\right)}^{4}\\ & -i\Omega_{d}\left(\hat{a}-{\hat{a}}^{†}\right)\cos\omega_{d}t. \end{array} \end{array}$$

**Fig. 2.**
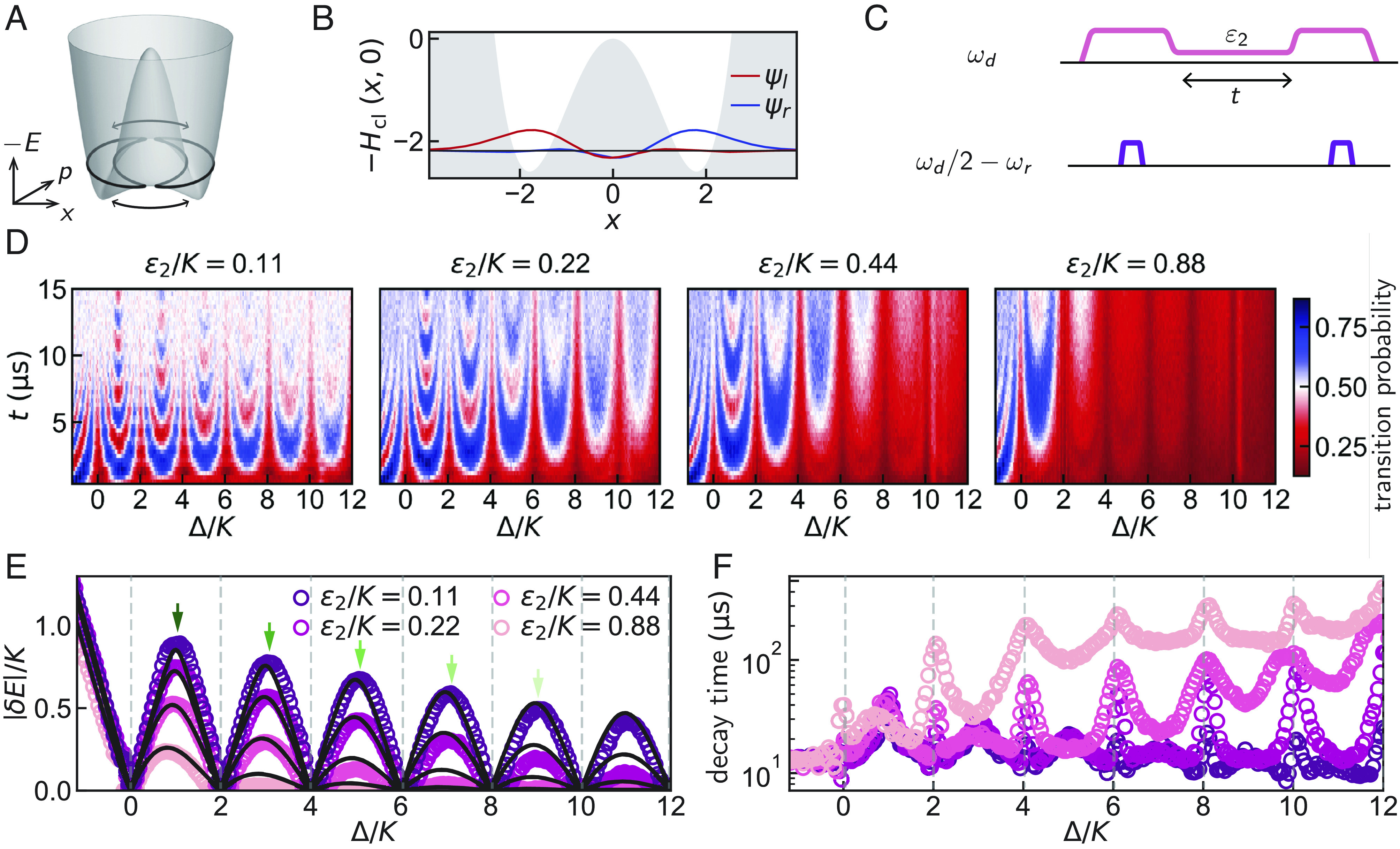
Tunnel-driven Rabi oscillations in the ground state manifold and their periodic cancellation. (*A*) Energy surface associated with Eq. **1** in the classical limit for Δ/K=3 and $\epsilon_{2}/K=0.11$. The orbits shown with black lines are obtained by semiclassical action quantization and represent the ground states (*SI Appendix*). Bidirectional arrows represent the two interfering WKB tunneling paths. (*B*) Cut of the energy surface in *A* at p=0 (*SI Appendix*). The classically forbidden region is marked in gray. The *Left* and *Right* localized wavefunctions are indicated in red and blue. (*C*) Pulse sequence for *D*. The pink line represents the squeezing drive at frequency $\omega_{d}$ and the purple lines represent the preparation and readout drives at frequency $\omega_{d}/2-\omega_{r}$. (*D*) Time-domain Rabi oscillation measurement of interwell tunneling probability (color) as a function of $\Delta^{\mathrm{bare}}$, taken here as Δ (*Text*), for $\epsilon_{2}/K=$ 0.11, 0.22, 0.44, and 0.88. The extracted tunneling amplitudes from *D* are shown as open circles in (*E*). The black lines in (*E*) correspond to the transition energy between the lowest eigenstates obtained from an exact diagonalization of Eq. **1**. A comparison of the extracted tunneling rate with a semiclassical WKB calculation is presented in *SI Appendix*. Green arrows in *E* denote the condition for constructive interference of tunneling and correspond to the measurements shown in Fig. 3. We extract the value of the Kerr coefficient *K* from this data and note that it is consistent, within experimental inaccuracies, with an independent saturation spectroscopy measurement of the Fock qubit in the absence of the squeezing drive (*SI Appendix*). (*F*) Decay time of the tunnel-driven Rabi oscillations for different values of Δ and $\epsilon_{2}$ in *D*. Sharp peaks in the decay time are clearly visible for Δ/K=2m, *m* being a nonnegative integer.

The squeezing of the oscillator can be understood as emerging from the down-conversion of one drive excitation into two oscillator excitations (Fig. 1*D*) and its conjugate process. To make this interaction resonant, the drive is configured so that its second subharmonic $\omega_{d}/2$ lies in the vicinity of the SNAIL transmon resonance at $\omega_{a}=\omega_{o}+3g_{4}-20g_{3}^{2}/3\omega_{o}+O\left(g_{3}^{3}/\omega_{o}^{2}\right)$. Thus, taking into account the quantum correction to the small oscillation frequency of the oscillator is important. For our device, we measure $\omega_{a}/2\pi =6.035\mathrm{GHz}$. The squeezing drive amplitude in Eq. **1** is related to the oscillator nonlinearity in Eq. **3** as $\epsilon_{2}=g_{3}\Pi$, where $\left|\Pi |=\right|\Omega_{d}\omega_{d}/\left(\omega_{d}^{2}-\omega_{o}^{2}\right)|$ is the linear response of the oscillator to the drive (20), and $\epsilon_{2}$ has been taken to be real-valued without loss of generality. Furthermore, the Kerr coefficient in Eq. **1** is related to the oscillator’s nonlinear coefficients as $K=10g_{3}^{2}/3\omega_{o}-3g_{4}/2+O\left(g_{3}^{3}/\omega_{o}^{2}\right)$ (20). In our experiment, we measure it to be K/2π=316.8kHz (Fig. 2). By directly measuring *K* and the $\omega_{a}$ as a function of the external magnetic flux, we can fit the nonlinear constant of our sample using the model for a SNAIL-array in ref. 10. Since the model has many free parameters that we can only infer from design, our best estimate for the nonlinear coefficient is $g_{3}/2\pi =3\times \left(-5\right)$ MHz ±50% and $g_{4}/2\pi =4\times \left(-32\right)$ kHz ±50%.

Note that this value of the Kerr coefficient is about three orders of magnitude lower than a regular transmon (21). This intentional design choice (10) avoids the effect of nonlinear resonances which are well-known to plague transmon (22–24) while retaining the sought-after low-order squeezing process. Thus, in Eq. **1** we tune $\epsilon_{2}$ and Δ independently with the amplitude and frequency of the squeezing drive. Furthermore, since *K* only provides an overall scale factor, we have independent real-time control of all the parameters of Eq. **1**. Due to the relatively small *K* compared to a standard transmon (21), our experiment has a negligible ac Stark shifts for $\epsilon_{2}/K≲1$, so that in this regime $\delta^{\mathrm{ac}}/K≲1\%$. Therefore, in this regime, Δ can be approximated by $\Delta^{\mathrm{bare}}=\omega_{a}-\omega_{d}/2$. To complete the characterization of our SNAIL transmon, we measure the single-photon lifetime of the undriven SNAIL transmon is $T_{1}=20$ µs and the Ramsey coherence between its lowest-lying eigenstates is $T_{2R}=3.8$ µs.

The readout is facilitated by a Beryllium Copper pin inserted into the cavity, which serves as the readout port and defines the linewidths of the readout resonator and Purcell filter. In our experiment, we measure the readout frequency to be $\omega_{r}/2\pi =8.5\mathrm{GHz}$. We perform readout by adding an additional drive at the difference frequency between the readout resonator and the second subharmonic of the drive. This second drive activates a parametric beam splitter that swaps the down-converted photon into the resonator leaking into our quantum-limited (25) readout chain (Fig. 1*E*). The linewidth of the resonator is κ/2π=0.4MHz. By this means we achieve over 99.5% single-shot, quantum nondemolition readout fidelity of which-well information. See ref. 10 for a more detailed characterization of the readout chain.

## Experimental Results.

We first experimentally demonstrate the cancellation of tunneling in the ground state manifold. In Fig. 2*A*, we show the classical limit of the energy surface, called the metapotential, associated with Eq. **1** for $\Delta /K=3,\epsilon_{2}/K=0.11$, as a function of phase-space coordinates. The arrows under the two saddle points indicate the two WKB tunneling paths in between the two wells (11). At these saddle points, the momentum is nonzero. By contrast, for a massive particle moving in a quadratic + quartic potential, tunneling through the barrier is associated with only one path under the barrier maximum, corresponding to zero momentum. In the more elaborate situation of Fig. 2*A*, the two tunneling paths can interfere. In this case, oscillations accompany the decay of the wavefunction in the classically forbidden region. This interference can even lead to the coherent cancellation of the tunneling amplitude altogether. This is especially interesting since this may occur for finite barrier height, allowing the tunneling to be restored when the interference is constructive. Whether the interference is destructive or constructive is decided by a combination of the barrier height and the well-distance. This is illustrated in Fig. 2*B* where we show the wavefunctions corresponding to the ground state manifold. In the general case, that these are not the energy eigenstates but their even and odd superpositions, which are localized in the left and right wells. Importantly, in the classically forbidden region, marked in gray, oscillations accompany the expected decay of the wavefunctions (11). To observe coherent cancellation of tunneling in the ground state manifold, we prepare a localized well state and measure its tunneling probability as a function of time for different values of Δ and $\epsilon_{2}$. We present the measurement protocol in Fig. 2*C*. The preparation is done by rapidly turning on the squeezing drive until an amplitude of $\epsilon_{2}/K=8.7$ is reached. We subsequently wait $5T_{1}$ for the system to relax to its steady state in the presence of the squeezing drive and measure, in a quantum nondemolition (QND) manner, the quadrature containing the which-well information. This measurement projects the system into one of the wells. This readout protocol yields a stabilized fluorescence signal revealing the quadrature measurement outcome, the squeezing drive sustaining the circuit oscillation. After the preparation, we adiabatically lower the squeezing drive amplitude in a duration 1.6μs≳π/K.^*^ The depth of the wells, which increases with $\epsilon_{2}/K$ (*SI Appendix*), is then reduced so that the tunnel effect becomes observable. We then wait for a variable amount of time before adiabatically raising the squeezing drive amplitude to its initial value. Finally, we measure which well the system has adopted.

The data for this tunneling measurement is shown in Fig. 2*D*, where we interpret the oscillating color pattern as tunnel-driven Rabi oscillations. The transition probability shown in Fig. 2*D* is measured by preparing the system in one well and letting it evolve freely, as explained previously (see Fig. 2*C* for the experimental sequence). We then measured the probability as a function of the time the system have been left to evolve under the tunneling Hamiltonian. The frequency of this oscillation yields measurement of ground-state tunneling (11).

The periodic cancellation of tunneling at Δ/K=2m, where *m* is a nonnegative integer, is clearly visible as a divergence of the Rabi period. We extract the tunneling amplitude |δE| from our data by fitting the oscillation frequency with an exponentially decaying sinusoid and plot this frequency in Fig. 2*E*, where the data-point color corresponds to the value of $\epsilon_{2}$ (see *SI Appendix* for calibration of $\epsilon_{2}$). The black lines, obtained from an exact diagonalization of the static effective Hamiltonian Eq. **1**, correspond to the energy difference between levels in the ground state manifold. The cancellation of tunneling for the ground state manifold in a parametrically modulated oscillator was predicted by Marthaler and Dykman (11) where, using a semiclassical WKB method, the authors found that this multipath interference effect is due to, and accompanied by, oscillations of the wavefunction crossing zero in the classically forbidden region. Here, we find good agreement between our experiment and their WKB prediction (*SI Appendix*). Note that, across the zero of the tunneling amplitude, the bonding and antibonding superposition of well states alternate as the ground state. Specifically, for Δ/K=4m+1, the ground state is the bonding superposition of well states (*SI Appendix*). In Fig. 2*F*, we further plot the extracted decay time of the tunneling oscillations as a function of Δ, and find sharp peaks when Δ/K=2m, besides an overall continuous increase of the decay time with Δ and $\epsilon_{2}$. The peaks at Δ/K=2m arise from the degeneracies in the excited state spectrum at this condition and are discussed later in the text.

Importantly, the dynamics of the two-level system in Fig. 2*D* suggest a type of bosonic encoding of information that we call the Δ-Kerr-cat qubit. The north and south poles of the corresponding Bloch sphere, a generalization of the Δ=0 one (7, 9, 10), is defined by the cat states formed by the lowest pair of eigenstates of Eq. **1**. In this picture, a tunnel-Rabi cycle in Fig. 2*D* for a fixed Δ/K≠2m corresponds to a travel along the equator. For Δ/K=2m, this travel is prohibited. Note that when Δ/K=2m+1, the tunneling amplitude is maximum and is first-order insensitive to fluctuations of Δ.

**Fig. 3.**
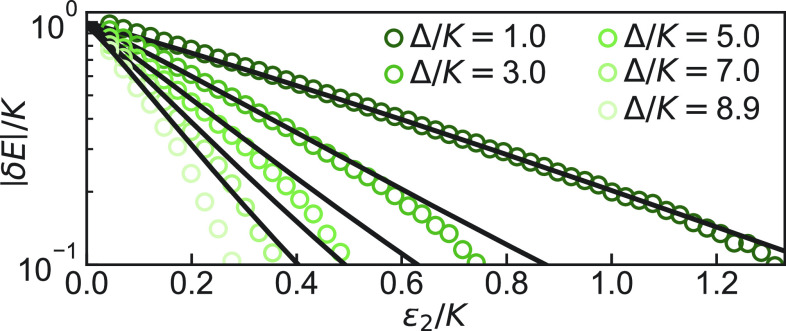
Exponential reduction of tunnel splitting as a function of $\epsilon_{2}$ in the ground state manifold. Extracted tunnel splitting (open circles) for the first five local maxima in Fig. 2*E* as marked by the color-coded arrows. Experimental sequence as in Fig. 2*E*. For the raw color data, see *SI Appendix*, Fig. 3. Black lines are obtained from a Hamiltonian diagonalization of Eq. **1** with no adjustable parameters. For comparison with a semiclassical WKB calculation, *SI Appendix*. Note that for small tunneling amplitude, dissipation plays a relevant role and the Hamiltonian model used here is insufficient.

From Fig. 2*E*, we also see that, besides the discrete cancellation of tunneling at Δ/K=2m, tunneling in the ground state manifold is overall continuously reduced with both Δ and $\epsilon_{2}$. This reflects the well-known symmetry of the double well, which is broken by tunnel coupling. The approximate symmetry is restored with increasing Δ and $\epsilon_{2}$ because both parameters explicitly control the barrier height and thus exponentially control the tunneling amplitude |δE|. Theory predicts that the larger the detuning Δ, the faster the tunneling reduction with the squeezing drive amplitude $\epsilon_{2}$ (*SI Appendix*). We have observed this effect by measuring the tunneling amplitude as a function of $\epsilon_{2}$ for different constructive tunneling conditions corresponding to Δ/K=2m+1. The data are presented in Fig. 3. The exponential insensitivity, around Δ=0, to fluctuations of Δ due to a noisy $\omega_{a}$, as a function of $\epsilon_{2}$, was predicted and thus proposed as a resource for quantum information (7). This insensitivity was a key motivation for realizing the Kerr-cat qubit (9). The insensitivity of the ground state manifold to detuning as a function of $\epsilon_{2}$ is directly observed here. Note from Fig. 2*E* that for Δ<0, in the parameter regime $\epsilon_{2}/K<1$, the tunneling amplitude |δE| is weakly dependent on $\epsilon_{2}$, whereas for Δ>0, it is strongly dependent on $\epsilon_{2}$. This weak dependence for Δ<0 is expected since the barrier height vanishes for small values of $\epsilon_{2}/K$.^†^ Our finding shows that new operating points at even, positive values of Δ/K increase the resilience of ground-state qubit encoding to detuning-like noise.

**Fig. 4.**
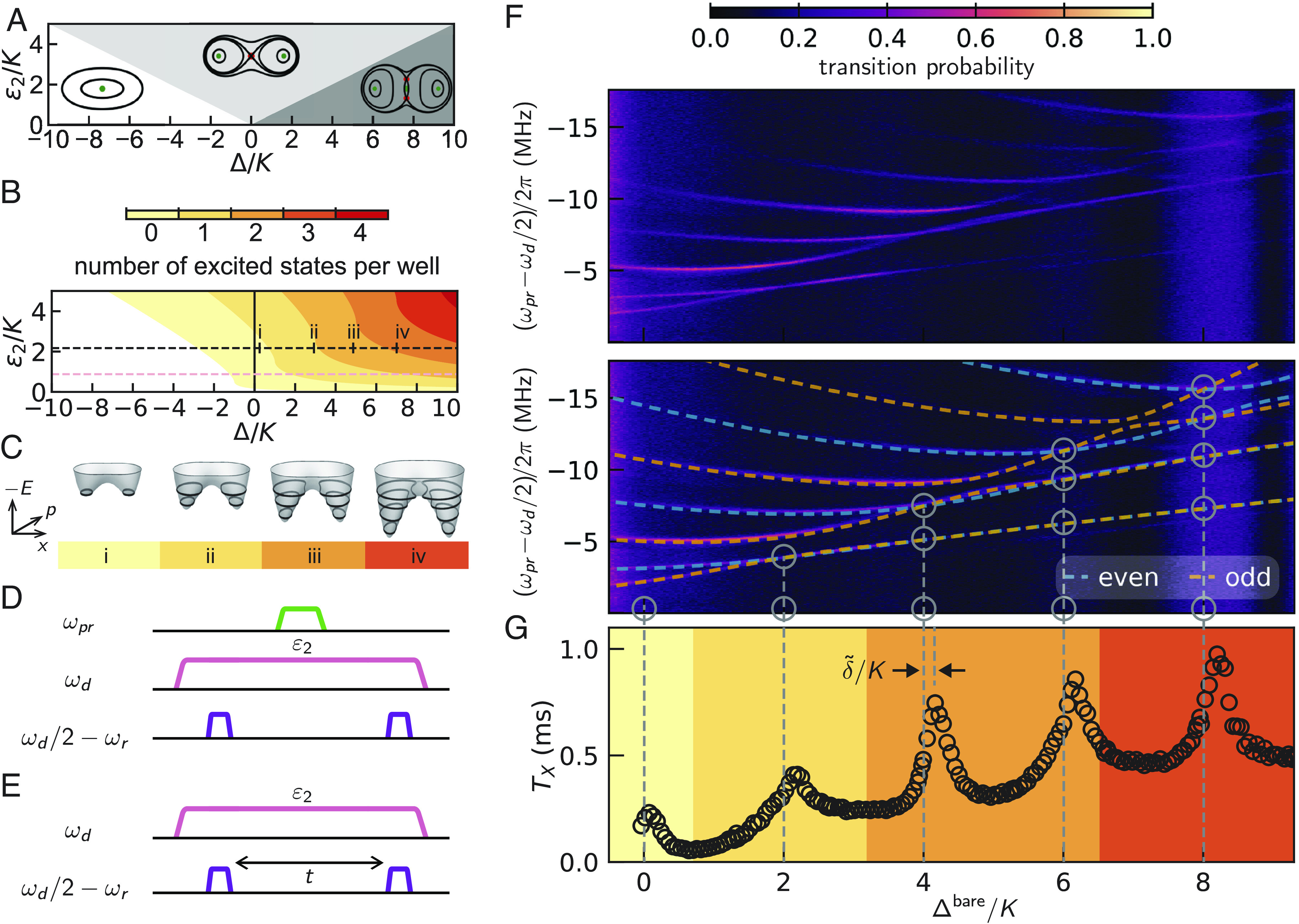
Spectroscopic measurements of coherent and periodic cancellation of tunnel splitting in the excited state spectrum. (*A*) Classical phase diagram for the Kerr oscillator with parametric squeezing, also called the period-doubling bifurcation diagram. (*B*) Quantum phase diagram to count in-well excited states. Colors represent contours of constant action on the energy surface associated with Eq. **1**. The dashed pink line corresponds to $\epsilon_{2}/K=0.88$, the maximum value of squeezing drive amplitude in Fig. 2. The dashed black line corresponds to $\epsilon_{2}/K=2.17$, the value of squeezing drive amplitude used in Fig. 3 *F* and *G*. (*C*) Energy surfaces for $\epsilon_{2}/K=2.17$ and i) Δ/K=0.5, ii) Δ/K=3, iii) Δ/K=5, and iv) Δ/K=7. Bohr-like obits are indicated as black curves (see *SI Appendix* for more details). (*D*) Pulse sequence for (*F*). The green line represents the weak spectroscopic probe tone at frequency $\omega_{\mathrm{pr}}$. The pink line represents the squeezing drive at frequency $\omega_{d}$ and the purple lines represent the preparation and readout drives at frequency $\omega_{d}/2-\omega_{r}$. (*E*) Pulse sequence for (*G*). (*F*) (*Upper* panel) Frequency-domain measurement of well-transition probability (color) via excited states as a function of Δ for $\epsilon_{2}/K=2.17$. The power of the perturbative spectroscopic probe is increased as $\omega_{\mathrm{pr}}$ is decreased to compensate for the lower matrix element connecting the ground state with the higher excited levels, yet is kept weak enough to preserve the parity conservation rules of Eq. **1**. (*F*) (*Lower* panel) Dashed lines plotted on *Top* of experimental data (same as in *Upper* panel) correspond to transition energies obtained by performing an exact diagonalization of Eq. **1** with no adjustable parameters. The Kerr coefficient is calibrated via time-domain measurements in Fig. 2*E*. (*G*) Measured well-switching time under incoherent environmental-induced evolution as a function Δ for $\epsilon_{2}/K\approx 2.17$. Background color in (*G*) marks the number of excited states per well following semiclassical orbit quantization.

Moving to the pairs of excited states above the ground state manifold, do they also present observable degeneracies as a function of Δ/K? In order to deepen our understanding of this problem, we first examine the classical energy surface associated with Eq. **1** via the period doubling phase diagram (27) shown in Fig. 4*A*. In the classical limit (*SI Appendix*), the parameter space spanned by Δ/K and $\epsilon_{2}/K$ is divided by two phase transitions located at $\Delta =\pm 2\epsilon_{2}$. The different phases are characterized by the number of stable nodes (attractors) in the metapotential referred to later as the single-, double-, and triple-node phases. These phases correspond to different metapotential topologies. We show them as contour line *Insets* in Fig. 4*A*, representing classical orbits. The single-node phase occurs for $\Delta <-2\epsilon_{2}$, and presents only one well. For $\Delta \geq -2\epsilon_{2}$, the oscillator has bifurcated and the classical metapotential acquires two wells. In the presence of dissipation, these wells house stable nodes. The emergent ground state manifold has been exploited, for Δ=0, in the Kerr-cat qubit (9, 10). In the interval $-2\epsilon_{2}\leq \Delta <2\epsilon_{2}$, an unstable extremum (saddle point) appears at the origin. For $\Delta \geq 2\epsilon_{2}$, the saddle point at the origin splits into two saddle points and an attractor reappears at the origin. The barrier height of the classical metapotential is given by ${\left(\Delta +2\epsilon_{2}\right)}^{2}/4K$ in the double-node phase and by $2\epsilon_{2}\Delta /K$ in the triple-node phase (*SI Appendix*). To count the number of excited states that have sunk under the barrier, we further introduce in Fig. 4*B* a semiclassical phase diagram of the squeeze-driven Kerr oscillator. Following the Einstein-Brillouin-Keller method, which generalizes the notion of Bohr orbits, we quantize the action enclosed in the metapotential well below the height of the barrier and obtain the number of in-well excited states. In Fig. 4*C*, we present the corresponding orbits in the energy surface for a fixed value of $\epsilon_{2}/K=2.17$ and four values of Δ/K. We validate this simple, semiclassical picture with a fully quantum mechanical calculation of the Wigner functions of localized states in the ground and excited state manifold (*SI Appendix*). It is clear from this analysis that, by increasing $\epsilon_{2}$ and Δ, and therefore the barrier height, not only the ground state manifold but even the excited state manifolds become progressively ensconced in the wells, and we thus expect the tunneling between the wells to be drastically reduced.

Besides the overall continuous reduction of tunneling, the excited state manifold of the squeeze-driven Kerr oscillator experiences a discrete cancellation of tunneling when Δ/K=2m. Since the squeezing interaction preserves photon parity, levels belonging to the even and odd sector of the Kerr Hamiltonian remain decoupled and repeatedly cross at values of Δ/K corresponding to even integers. This braiding induces m+1 perfect degeneracies at Δ/K=2m. Moreover, the corresponding eigenstates have a closed-form expression in the Fock basis. Remarkably, these features are independent of the value of $\epsilon_{2}$, reflecting a particular, unappreciated symmetry of our Hamiltonian Eq. **1** (*SI Appendix*).

Both the discrete cancellation and the overall continuous reduction of tunneling now in the excited state manifold of the squeeze-driven Kerr oscillator is accessed by performing spectroscopy measurements as a function of Δ, which we show in Fig. 4*F* for $\epsilon_{2}/K=2.17$. The measurement protocol is shown in Fig. 4*D*. We prepare a localized well state in a manner that is similar to the protocols of Figs. 2 and 3. To locate the frequency of the excited states, we apply a probe tone at variable frequency in the vicinity of the SNAIL transmon resonance $\omega_{a}$ and measure the well-switching probability. When the probe is resonant with a transition to a state close to the barrier maximum, this probability is increased. The experimental results are shown in Fig. 4*F*. The colored dashed lines (orange and blue) in the lower panel are obtained from an exact diagonalization of the static effective Hamiltonian Eq. **1** with no adjustable parameters. The crossings of levels are marked with circles. The data also shows that the level crossings are accompanied by a continuous reduction of the braiding amplitude with Δ. The corresponding reduction of the tunnel splitting is the manifestation associated with a generic double-well Hamiltonian, while the braiding reflects interference specific to our particular Hamiltonian, resulting from its underlying driven character. The level of experimental control achieved allows us to observe in this data the joint presence of the exact discrete symmetry and the approximate continuous symmetry in our bosonic system.

An important consequence of the cancellation of tunneling in the excited state spectrum is the periodic enhancement of the well-switching time under incoherent environment-induced evolution. This time scale corresponds to the transverse relaxation time, $T_{X}$, of a new bosonic qubit: a Δ-variant of the Kerr-cat qubit (7, 26) as mentioned earlier. To measure $T_{X}$, we prepare a localized well state by measurement, and wait for a variable amount of time before measuring the which-well information. We show the pulse sequence in Fig. 4*E*. We obtain $T_{X}$ by fitting a decaying exponential function to the measured well-transition probability for each value of Δ and plot the result in Fig. 4*G*. Note that we have chosen the squeezing drive amplitude identical to that of Fig. 4*F*, as $\epsilon_{2}/K=2.17$. Around values of Δ/K corresponding to even integers, the variation of $T_{X}$ presents sharp peaks. The location of the peaks corresponds to the degeneracy condition in the excited state spectrum, associated with coherent cancellation of tunneling and the blocking of noise-induced well-switching pathways via the excited states. The systematic right-offset $\tilde{\delta }/K$ of each peak from an even integer, is 15%. About 5% can be attributed to the ac Stark shift $\delta^{\mathrm{ac}}$ for the photon number corresponding to $\epsilon_{2}$, given the accuracy of our knowledge of the experimental parameters. We do not have a firm explanation for the remaining 10%, but we suspect higher-order terms in our static effective Hamiltonian. Note that this explanation is still compatible with the perfect alignment of the cancellation points with even integers in Fig. 2*F* for $\epsilon_{2}/K<1$, since for that case the ac Stark shift is negligible. Note also that this offset could provide access, within experimental accuracy, via the ac Stark shift, to the nonlinear coefficients of Eq. **2**.

The data in Fig. 4*G* also shows that the discrete peaks are accompanied by a monotonic baseline increase, a direct manifestation of the overall continuous tunneling reduction in the spectrum versus Δ. The background colored stripes represent the number of in-well excited states found via the action quantization method discussed above and in *SI Appendix*. Continuing with this semiclassical picture, we interpret the slowdown in the growth of $T_{X}$ for Δ/K≳5 as resulting from the increase of the barrier height as one crosses over from the double-node, where the barrier height $\propto {\left(\Delta +2\epsilon_{2}\right)}^{2}$, to the triple-node phase, where the barrier height $\propto \Delta \epsilon_{2}$. Indeed, this is the quantum manifestation of the classical phase transition from the double-node to the triple-node phase. The heights and widths of the peaks should be quantitatively compared with theory, but this subject is beyond the scope of this article.

**Fig. 5.**
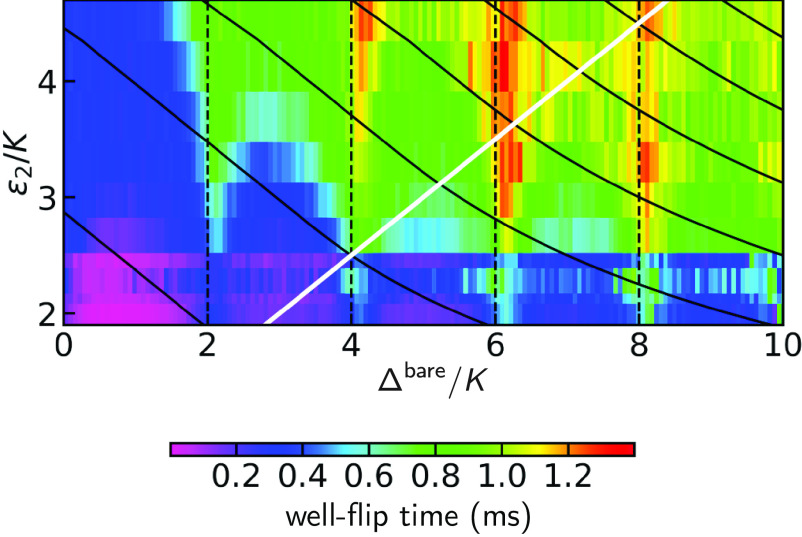
Color plot of $T_{X}$ as a function of Δ/K and $\epsilon_{2}/K$. The white line marks the transition from a two-node to a three-node metapotential. Black solid lines mark contours of constant barrier height. Increasing both Δ/K and $\epsilon_{2}/K$ yields fastest enhancement in $T_{X}$ as predicted by Fig. 4*B*. The additional enhancement by the coherent cancellation of excited state tunneling at Δ/K=2m stands out. The pulse sequence for the measurement is shown in Fig. 4*E*.

Thus, whether the theoretical framework is classical, semiclassical, or quantum, the predicted $T_{X}$ will increase with both $\epsilon_{2}$ and Δ. While $\epsilon_{2}$ and Δ contribute via the overall continuous reduction of tunneling (10), only Δ controls the discrete cancellation of tunneling. We verify this prediction by measuring $T_{X}$ while varying simultaneously both Hamiltonian parameters. We present the result of this experiment in Fig. 5. We further plot contours of constant barrier height in black, and the expected separation between the double-node and triple-node metapotential as a white line. We do not expect any sharp features along this line since the system lies deeply in the quantum regime. Following the gradient of the barrier height, one observes as expected the fastest gain in $T_{X}$, with a maximum of $T_{X}=1.3\mathrm{ms}$ for Δ/K=6 and $\epsilon_{2}/K=4$. Increasing the lifetime by increasing $\epsilon_{2}$ presents limitations, since strong drives are known to cause undesired effects in driven nonlinear systems (see refs. 23 and 28 and *SI Appendix*).

One could argue that Δ=0 provides an important factorization condition that guarantees that the ground state manifold is spanned by exact coherent states (see ref. 7 and *SI Appendix*). Indeed, this is an asset for quantum information, since these states are eigenstates of the single-photon loss operator $\hat{a}$ (29). However, this desirable property is traded for the advantages discussed earlier when Δ/K=2m, m≥1. Even if the Δ-variant of the Kerr-cat qubit suffers from quantum heating and quantum diffusion (14, 30–33) at zero temperature resulting from the squeezed nature of its ground states, these effects are small (16) and, as we show in the experiments reported here and in ref. 10, the well-states of the Kerr-cat live longer than its Δ=0 parent, even at finite temperature.

## Discussion

Although quantum tunneling was discovered nearly a century ago (34) and observed since in a variety of natural and synthetic systems, the treatment of tunneling is usually limited to the ground states of the system and has rarely been discussed in the literature for excited states, as we elaborate in the following survey. The phenomenology of ground state tunneling has been studied in cold atoms (35) in three-dimensional optical lattices (36), optical tweezers (37), ion traps (38) and in quantum dots (39). In Josephson tunnel circuits, quantum tunneling of the phase variable was first observed by Devoret et al. (40) and since then exploited in several other experiments (41). Furthermore, the tunnel effect has been involved in quantum simulation (42), in Floquet engineering of topological phases of matter and to generate artificial gauge fields with no static analog (43, 44). The quantum interference of tunneling for the ground states of a large spin system was measured previously in a cluster of eight iron atoms by Wernsdorfer and Sessoli (45) (see also ref. 46).

Weilinga and Milburn (13) first identified that the quantum optical model in Eq. **1** exhibits ground state tunneling for a particular value of Δ. Marthaler and Dykman (11, 14) developed a WKB treatment for a range of the Δ parameter, and predicted that, for this model, the tunnel splitting of the ground state manifold crosses zero periodically and is accompanied by oscillation of the wavefunction in the classically forbidden region.

Our work is an experimental realization of the longstanding theoretical proposals of the last paragraph. It is similar, but different, to the phenomenology of the “coherent destruction of tunneling”, discovered theoretically by Grossmann et al. (47) and observed experimentally in cold atoms (48, 49). Indeed, the dynamical tunneling in our experiment is in sharp contrast with photon-assisted or suppressed tunneling in weakly driven double-well potentials. First, our tunneling is completely dynamical, i.e., the tunneling barrier vanishes in the absence of the drive and the drive operates in a completely different regime of frequency and it belongs to a different class of physical effects: Grossmann et al.’s theory (47) requires a drive resonant with the first two levels within the wells, here $∼4\epsilon_{2}\ll \omega_{d}$. Second, and most importantly, our work extends the coherent cancellation of tunneling to all the excited states in the well. The periodic resonance condition Δ/K=2m, shared for the m+1 first pairs of excited levels, is independent of the drive amplitude. Remarkably, under this multistate resonance condition, the first 2(m+1) oscillator states have a closed-form expression in the Fock basis (*SI Appendix*). We further emphasize that the dynamical tunneling in our work is distinct from chaos-assisted dynamical tunneling (50) observations made in ultracold atoms over three decades ago (50, 51); remarkably our strongly driven nonlinear system remains integrable and well described by a static effective model. Our work corresponds to the experimental finding of the exact simultaneous cancellation of the tunnel splitting for the ground and excited states. Our data featuring the incoherent dynamics can be qualitatively modeled by a Lindbladian treatment that we present in *SI Appendix*, yet more research on the decoherence of driven nonlinear driven systems is needed to get a quantitative agreement (see ref. 28).

As a resource for quantum information, the squeeze-driven Kerr oscillator for Δ=0, was identified in theory proposals by Cochrane et al. (8) for trapped ions in 1999 and Puri, Boutin, and Blais (7) for superconducting circuits in 2017 due to its exponential resilience to low frequency noise and was proposed for a bosonic code. The theory of bistability for the non-zero Δ case was studied by Zhang and Dykman in (12) and Roberts and Clerk in (15), and their qubit operation was investigated in (16). Our work demonstrates this bistability experimentally through the lifetime peaks in Fig. 4*G* and explains the peaks as a fingerprint of the observed spectral degeneracies in Fig. 4*F*. Furthermore, the resilience to noise in the case of nonzero Δ is demonstrated through Figs. 2*E* and 3.

## Conclusion

We have observed multiple degeneracies between pairs of states in a quantum double-well system, resulting from the interplay of quantum tunneling and quantum interference. Our results provide experimental evidence of the cancellation of tunneling due to interference in the classically forbidden region (11).

Our work showcases the tunability of these degeneracies in number and the ability to rapidly activate or deactivate them. Furthermore, we have identified the drive frequency as a critical control parameter, governing not only a discrete exact symmetry in Eq. **1**, manifested as exact degeneracies as a function of Δ, but also a continuous approximate symmetry as a function of $\epsilon_{2}$ that leads to an overall exponential reduction of tunnel splitting in both ground and excited states of our oscillator. This degree of quantum control a significant reduction of incoherent well-flip dynamics, leads to enabling a protected cat-qubit: the Δ-Kerr-cat qubit. Our demonstration of the continuous Z-gate (17, 52) adds valuable capability to the single qubit gate-set for cat qubits, offering tools for quantum computation (7, 9, 10, 15, 29, 52–56).

After our experiments were performed, we learned that the degeneracies in our squeeze-driven Kerr oscillator were studied theoretically by our colleagues in the QUANTIC group in INRIA, Paris (16).

## Supplementary Material

Appendix 01 (PDF)

## Data Availability

The data that support the findings of this work are openly available in Zenodo at ref. 57. All other data are included in the manuscript and/or *SI Appendix*.

## Footnotes

^*^Note that this adiabaticity condition pertains to the gap between the ground and first excited pair of states. We do not need to be adiabatic with respect to the two tunnel split states within the ground state manifold since they have opposite parity and the parity preserving squeezing drive will not couple them.

^†^In the absence of dissipation, the metapotential acquires two wells as soon as $\epsilon_{2},\Delta >0$, i.e., there is no threshold for bifurcation of the driven oscillator. In our quantum experiment, this threshold is finite but is, relatively speaking, extremely small since and is set by $\epsilon_{2}^{2}>\left(\Delta^{2}+T_{1}^{-2}/4\right)/4$ (see ref. 26).
