# Supplementary material for: A driven Kerr oscillator with two-fold degeneracies for qubit protection
Source: Proc Natl Acad Sci U S A. 2024 Jun 5;121(24):e2311241121. doi: 10.1073/pnas.2311241121 (PMC11181142; doi:10.1073/pnas.2311241121)
Supplement: Supplementary file 1 — Appendix 01 (PDF) [file pnas.2311241121.sapp.pdf]

Supplemental Material:
A driven Kerr oscillator with two-fold degeneracies for qubit protection

Jayameenakshi Venkatraman,* Rodrigo G. Cortiñas,† Nicholas E. Frattini,‡ Xu Xiao, and Michel H. Devoret§
Department of Applied Physics and Department of Physics, Yale University, New Haven, CT 06520, USA
(Dated: March 16, 2024)

The Supplemental text is organized as follows. In Section I, we detail the experimental calibration of Hamiltonian parameters in Eq. (2) of the main text; specifically, in Section I we present a calibration of the squeezing drive ϵ_2 and in Section II, we present a measurement of the Kerr coefficient K. In Sections III and IV, we present further experimental results supplementing Figures 2 and 4 in the main text.

In Section V and the following sections, we switch gears and detail our theoretical models. First, in Section V we formally introduce the notation employed throughout this work. We then present in Section VI two well-known squeeze-driven Kerr oscillator Hamiltonians that were introduced in the literature and comment on the relationships between them. In Section VII, we introduce the operator and phase space formulation of our particular squeeze-driven Kerr oscillator effective Hamiltonian and discuss its classical limit.

In Sections VII A, VIII and IX, we discuss distinct properties of this Hamiltonian and its eigenstates. Specifically, in Section VII A, we discuss the structure of lowest pair of well-localized wavefunctions for different Hamiltonian parameter configurations and distinguish them from those of an ordinary quadratic + quartic double-well potential. We present our semiclassical analyses, namely a WKB analysis of the tunnel splitting in Section VIII A and an overview of action quantization in Section VIII B to discuss the construction of quantized orbits. We discuss in Section IX the robustness of the degeneracies in the squeeze-driven Kerr oscillator. In Section X, we present a simple Lindblad model to capture the qualitative features of the experimentally measured transverse relaxation lifetimes T_X of the Δ-variant of the Kerr-cat qubit.

Finally, in Section XI we present a self-contained tutorial and a concise introduction to the phase space formulation of quantum mechanics.

I. CALIBRATING THE SQUEEZING DRIVE AMPLITUDE ϵ_2

In this section, we present a measurement that provides an independent calibration of the squeezing drive amplitude ϵ_2. The pulse sequence is the following: We turn on the squeezing drive at $\Delta = 0$, for a variable amount of time t during which we also turn on a Rabi drive at amplitude ϵ_x and frequency $\omega_d/2 = \omega_a$. The squeezing drive stabilizes the Schrödinger cat states with well-defined parity, and the Rabi drive induces an oscillation in this cat-qubit. We perform this experiment for different values of ϵ_2 and measure $\hat{X} = |\mathcal{C}^+\rangle\langle\mathcal{C}^-| + |\mathcal{C}^-\rangle\langle\mathcal{C}^+|$, where $|\mathcal{C}^\pm\rangle$ are the Schrödinger cat states. This protocol was introduced in [1, 2] and we refer the reader to these works for further details. The result of our experiment is shown in Figure S1**A**. From this experimental data, we extract a Rabi oscillation frequency Ω_x that is related to the amplitude of the Rabi drive as $\epsilon_x = \Omega_x(\epsilon_2 = 0)/2$. The photon-number at $\Delta = 0$ $|\alpha|_0^2$ is related to ϵ_x and Ω_x as $|\alpha|_0^2 = \Omega_x^2/16\epsilon_x^2$ [1, 2]. In Figure S1**B**, we plot the experimental data and fit for the extracted photon-number as a function of the digital control amplitude (DAC). With this result, we have a calibration of ϵ_2 as a function of the digital control amplitude (DAC) controlling the squeezing drive.

II. MEASURING THE KERR COEFFICIENT K

In this section, we detail a measurement of the Kerr coefficient K via saturation spectroscopy of the SNAIL transmon. This measurement is performed in the absence of the squeezing drive. In the following text, the letters g, e, and f index the ground, first excited, and second-excited states of the SNAIL transmon oscillator. In Figure S2, we plot the response of the readout as a function of a probe tone, whose frequency is ω_{pr}, and which we vary around the

*Electronic address: `jayavenkat@ucsb.edu`; these two authors contributed equally.
Present address: Department of Physics, Broida Hall, University of California, Santa Barbara, Santa Barbara, CA 93106, USA
†Electronic address: `rodrigo.cortinas@yale.edu`; these two authors contributed equally.
‡Present address: Nord Quantique, Sherbrooke, QC J1J 2E2, Canada
§Electronic address: `michel.devoret@yale.edu`

Typeset by REVTEX

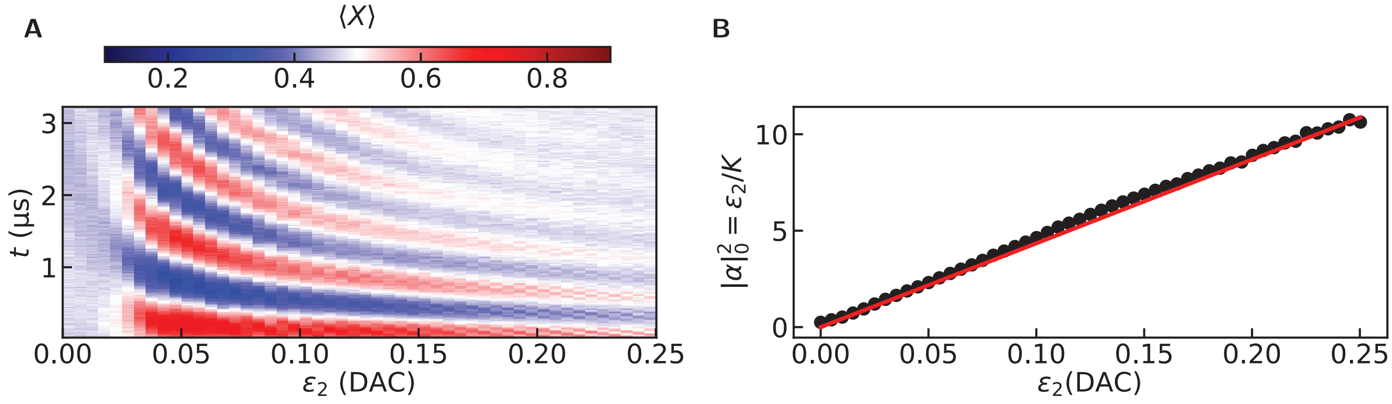

Figure S1: **Calibrating ϵ_2 with cat-Rabi oscillations. A** Color plot of $\langle X\rangle$ as a function of the the digital control amplitude (DAC) controlling the squeezing drive ϵ_2 and duration of the Rabi drive. We find $\epsilon_x/2\pi = 144.93$ kHz using the relation between the Rabi amplitude and Rabi frequency for $\epsilon_2 = 0$, $\epsilon_x = \Omega_x(\epsilon_2 = 0)/2$. A plot of $|\alpha|_0^2 = \epsilon_2/K = \Omega_x^2/16\epsilon_x^2$ [1, 2] as a function of ϵ_2 in DAC units. A line fit gives us a calibration of $|\alpha|_0^2 = \epsilon_2/K$ as a function of the digital control amplitude (DAC) controlling the squeezing drive.

ge transition frequency of the SNAIL transmon oscillator ω_a corresponding to $\epsilon_2 = 0$. When the probe tone excites the oscillator, the readout signal due to the dispersive coupling [3] changes. The two dips in Figure S2, from left to right, correspond to a two-photon transition that excites the oscillator from g to f and to a resonant excitation of the oscillator from g to e respectively. The $gf/2$ and ge resonances are located at $(\omega_a - K)/2\pi$ and $\omega_a/2\pi$ respectively. Fitting the peaks and subtracting their locations yields a value of $K/2\pi = (329.73\pm4.30)$ kHz. This value is consistent with the value of $K/2\pi = 316.83$ kHz, where the latter is extracted from Figure 1**E** in the main text and is the value for K used throughout the article.

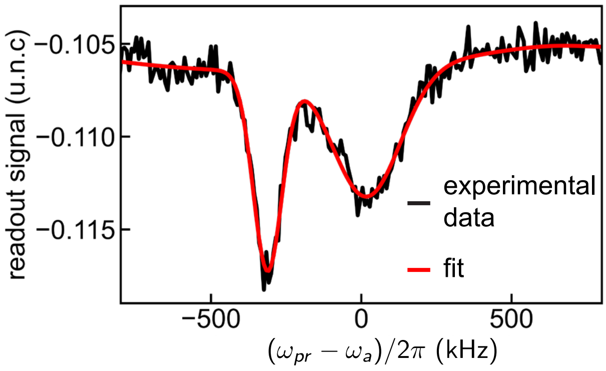

Figure S2: Readout response as a function of the frequency of the saturation (probe) tone. The two readout signal dips in black correspond, from left to right, to the $gf/2$ transition, which is expected to occur at $(\omega_a - K)/2\pi$ and to the ge transition of the SNAIL transmon, which is expected to occur at $(\omega_a)/2\pi$. Here, $gf/2$ refers to a transition induced by two photons from the probe. By fitting the experimental data, we find $K/2\pi = (329.73 \pm 4.30)$ kHz.

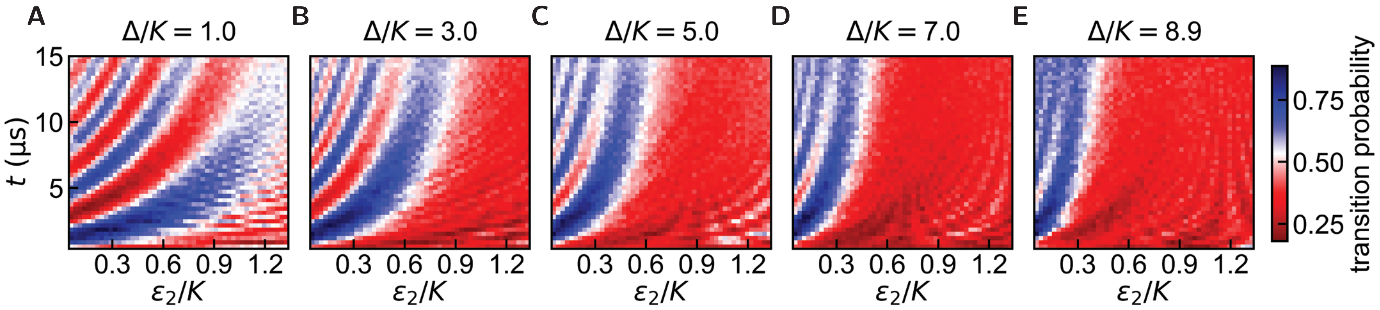

Figure S3: **Tunnel-driven Rabi oscillations in the ground state manifold and its exponential reduction as a function of ϵ_2; raw data.** The transition probability as a function of ϵ_2/K and time t for **A** $\Delta/K = 1$, **B** $\Delta/K = 3$, **C** $\Delta/K = 5$, **D** $\Delta/K = 7$, and **E** $\Delta/K = 9$ respectively. This corresponds to the condition of constructive interference of tunneling to occur. By progressively increasing ϵ_2, there is a clear overall continuous reduction of the tunnel-driven Rabi oscillations.

III. EXPONENTIAL REDUCTION OF TUNNELING WITH ϵ_2

In the main text, we claim that tunneling in the ground state manifold is overall continuously reduced with ϵ_2. The parameter ϵ_2 controls the barrier height, which is given as $(\Delta + 2\epsilon_2)^2/4K$ in the double-node phase and $2\Delta\epsilon_2/K$ in the triple-node phase. Moreover, continuing this reasoning, the larger the detuning, the faster the tunneling reduction as a function of ϵ_2. In Figure S3, we present raw data to further support this claim. We present the measurement protocol in Figure 1**C** of the main text and recall it for the sake of completeness. First, we prepare, by measurement, a steady-state localized in one of the wells. Following this, we adiabatically lower the squeezing drive amplitude ϵ_2. Lowering the value of ϵ_2 reduces the barrier depth, and thus the tunnel effect becomes observable. We then wait for a variable amount of time before adiabatically re-raising ϵ_2 to its initial value and finally do which-well readout.

In Figure S3, we present the measured transition probability as a function of ϵ_2 for **A** $\Delta/K = 1$, **B** $\Delta/K = 3$, **C** $\Delta/K = 5$, **D** $\Delta/K = 7$, and **E** $\Delta/K = 9$ respectively. It is clear from the data that the Rabi-frequency is overall continuously reduced with ϵ_2 and moreover, increasing Δ/K reduces the Rabi frequency further. We plot in Figure 2 of the main text the extracted tunneling amplitude $|\delta E|$ from our data by fitting the oscillation frequency with an exponentially decaying sinusoid. We find that the extracted tunneling amplitude is in excellent agreement with an exact diagonalization of the static effective Hamiltonian and in good agreement with a WKB prediction of the tunnel splitting within the expected regime of validity. See Figure S8 for more details.

IV. TRANSVERSE RELAXATION LIFETIME T_X MEASUREMENTS

In Figure S4, we plot the transverse relaxation lifetime T_X as a function of $\Delta^{\text{bare}} = \omega_a - \omega_d/2$ for different values of ϵ_2. Note that the photon-number at $\Delta = 0$ is given by $|\alpha|_0^2 = \epsilon_2/K$. Importantly, for large photon-numbers $\epsilon_2/K \gtrsim 6.5$, we see that the peaks in lifetime start plateauing and even dropping. This effect is not captured by an ordinary model of the Lindblad master equation as we discuss in Section X. The degradation of the T_1 with readout power has been observed for transmon qubits [5]. But other drive-induced effects such as multiphoton nonlinear resonances are present in transmons and disentangling these various sources of lifetime degradation is nontrivial [3, 5–8]. These spurious nonlinear resonances are largely absent in this our SNAIL conducting circuit for values of $\epsilon_2/K \lesssim 5$, thanks to negligible Kerr and stark shifts, but may plague our system for larger mean-photon numbers. Due to this reasoning, Δ might be a more effective knob to create states with large photon number [4]. Finally, the squeeze-driven Kerr oscillator provides a perfect platform to investigate lifetime degradation under drives.

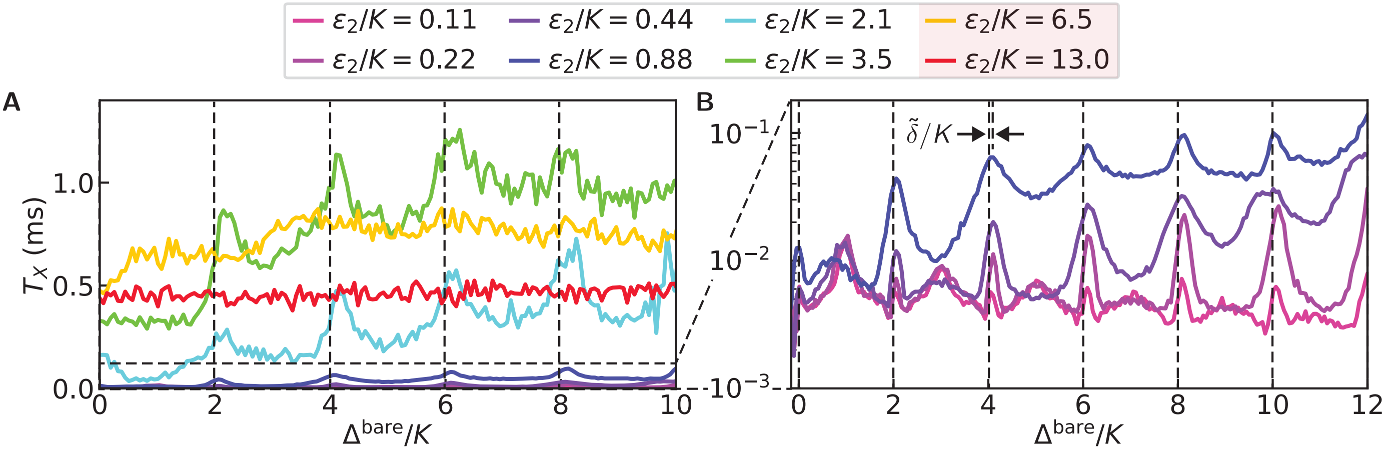

Figure S4: **Measurement of T_X as a function of $\Delta^{\rm bare} = \omega_a - \omega_d/2$ for representative values of squeezing drive amplitude ϵ_2.** The measurement protocol is shown in Figure 3**E** of the main text. We observe a degradation of T_X with increasing ϵ_2, as indicated by red boxes in the legend, and we show representative measurements here. On the other hand, we see no degradation of T_X with increasing Δ. This measurement indicates that Δ might be a more effective knob to increase T_X than ϵ_2 for cat-states with large photon-number [4]. **B** Low-power lifetime extracted from time-resolved measurements. The data is presented as Figure 1**F** in the main text.

V. NOTATION

In this work, we note $\hat{X}$ and $\hat{P}$ the *position*-like and *momentum*-like coordinates with $[\hat{X}, \hat{P}] = i\hbar$. We build the dimensionless quadratures by introducing the zero point spread of the coordinates as $X_{\rm zps}$ and $P_{\rm zps}$, respecting $X_{\rm zps}P_{\rm zps} = \hbar/2$. We further introduce the complex notation for the dimensionless quadratures as $\hat{a} = (\hat{X}/X_{\rm zps} + i\hat{P}/P_{\rm zps})/2$ and its conjugate operator $\hat{a}^\dagger$, where $[\hat{a}, \hat{a}^\dagger] = 1$ and introduce the rescaled phase space quadratures as $\hat{x} = \sqrt{\lambda/2}\hat{X}/X_{\rm zps} = \sqrt{\lambda/2}(\hat{a} + \hat{a}^\dagger)$ and $\hat{p} = \sqrt{\lambda/2}\hat{P}/P_{\rm zps} = -i\sqrt{\lambda/2}(\hat{a} - \hat{a}^\dagger)$, where $[\hat{x}, \hat{p}] = i\lambda$. These choices induce the definitions $x_{\rm zps} = p_{\rm zps} = \sqrt{\lambda/2}$. Conversely, we have $\hat{a} = (\hat{x} + i\hat{p})/\sqrt{2\lambda}$. At this point, λ is a dimensionless rescaling parameter. We will connect it with the Hamiltonian parameters later, while discussing the classical limit $(\lambda \to 0)$ of our system, and thereby give it physical significance. It is also useful to compare our results with those of [9], who have performed a WKB analysis of a driven oscillator. Thus, unless otherwise specified, λ should be taken equal to unity $\lambda = 1$.

For a mechanical oscillator with mass m and spring-constant k, the small-oscillation frequency is $\omega_o = \sqrt{k/m}$ and the impedance is $Z_o = 1/\sqrt{km}$. With this, we have $X_{\rm zps} = \sqrt{\hbar Z_o/2}$ and $P_{\rm zps} = \sqrt{\hbar/2Z_o}$. We further remark that there is a direct correspondence between the mechanical harmonic oscillator and a linear LC circuit oscillator [3, 10, 11] under the following relations. The mechanical position coordinate $\hat{X}$ corresponds to the circuit flux $\hat{\Phi}$, the mechanical momentum $\hat{P}$ corresponds to the circuit charge $\hat{Q}$, where $[\hat{\Phi}, \hat{Q}] = i\hbar$, the mechanical oscillator frequency $\omega_o = \sqrt{k/m}$ corresponds to the circuit oscillator frequency $\omega_o = 1/\sqrt{LC}$ and the mechanical oscillator impedance $Z_o = 1/\sqrt{km}$ corresponds to the circuit oscillator impedance $Z_o = \sqrt{L/C}$ which amounts to the identification of the mechanical mass m with the circuit capacitance C and the spring constant k with the inverse inductance $1/L$. The expressions for the zero point spreads are given by $\Phi_{\rm zps} = \sqrt{\hbar Z_o/2}$ and $Q_{\rm zps} = \sqrt{\hbar/2Z_o}$. In circuits, it is customary to introduce [3, 12] the reduced flux and charge coordinates: $\hat{\varphi} = \sqrt{\lambda}2\pi\hat{\Phi}/\Phi_0$ and $\hat{N} = \sqrt{\lambda}\hat{Q}/2e$ so that $[\hat{\varphi}, \hat{N}] = i\lambda$, where e is the charge quantum, and $\Phi_0 = h/2e$ is the magnetic flux quantum.[1] Their respective zero point spreads $\varphi_{\rm zps} = \sqrt{\lambda}2\pi\Phi_{\rm zps}/\Phi_0$ and $N_{\rm zps} = \sqrt{\lambda}Q_{\rm zps}/2e$, and are related to the rescaled complex coordinate operators by $\hat{\varphi} = \varphi_{\rm zps}(\hat{a}^\dagger + \hat{a})$ and $\hat{N} = -iN_{\rm zps}(\hat{a} - \hat{a}^\dagger)$ and $\varphi_{\rm zps}N_{\rm zps} = \lambda/2$. We summarize this notation in the following table

---

[1] Note that in this case the non-dimensionalization of variables is done by fundamental constants and not by linear properties of the oscillator. This comes at the price of a slight notation asymmetry over the reduced operators the electric and mechanical oscillators.

Mechanical oscillator	Circuit oscillator
$\hat{X};\ \hat{P}$	$\hat{\Phi};\ \hat{Q}$
$[\hat{X},\hat{P}]=i\hbar$	$[\hat{\Phi},\hat{Q}]=i\hbar$
$\omega_o=\sqrt{k/m}$	$\omega_o=1/\sqrt{LC}$
$Z_o=1/\sqrt{km}$	$Z_o=\sqrt{L/C}$
$X_{\rm zps}=\sqrt{\hbar Z_o/2}=\sqrt{\frac{\hbar}{2m\omega_o}}$; $P_{\rm zps}=\sqrt{\hbar/2Z_o}=\sqrt{\frac{\hbar m\omega_o}{2}}$ $\Rightarrow X_{\rm zps}P_{\rm zps}=\hbar/2$	$\Phi_{\rm zps}=\sqrt{\hbar Z_o/2}=\sqrt{\frac{\hbar}{2C\omega_o}}$; $Q_{\rm zps}=\sqrt{\hbar/2Z_o}=\sqrt{\frac{\hbar C\omega_o}{2}}$ $\Rightarrow \Phi_{\rm zps}Q_{\rm zps}=\hbar/2$
$\hat{a}=\frac{1}{2}\left(\frac{\hat{X}}{X_{\rm zps}}+i\frac{\hat{P}}{P_{\rm zps}}\right)$	$\hat{a}=\frac{1}{2}\left(\frac{\hat{\Phi}}{\Phi_{\rm zps}}+i\frac{\hat{Q}}{Q_{\rm zps}}\right)$
$\hat{X}=X_{\rm zps}\left(\hat{a}+\hat{a}^\dagger\right)$	$\hat{\Phi}=\Phi_{\rm zps}\left(\hat{a}+\hat{a}^\dagger\right)$
$\hat{P}=-iP_{\rm zps}\left(\hat{a}-\hat{a}^\dagger\right)$	$\hat{Q}=-iQ_{\rm zps}\left(\hat{a}-\hat{a}^\dagger\right)$
$\left[\hat{a},\hat{a}^\dagger\right]=1$	$\left[\hat{a},\hat{a}^\dagger\right]=1$
$\hat{x}=\sqrt{\frac{\lambda}{2}}\frac{\hat{X}}{X_{\rm zps}}=x_{\rm zps}\left(\hat{a}+\hat{a}^\dagger\right)$	$\hat{\varphi}=\sqrt{\lambda}2\pi\frac{\hat{\Phi}}{\Phi_0}=\varphi_{\rm zps}\left(\hat{a}+\hat{a}^\dagger\right)$
$\hat{p}=\sqrt{\frac{\lambda}{2}}\frac{\hat{P}}{P_{\rm zps}}=-ip_{\rm zps}\left(\hat{a}-\hat{a}^\dagger\right)$	$\hat{N}=\sqrt{\lambda}\frac{\hat{Q}}{2e}=-iN_{\rm zps}\left(\hat{a}-\hat{a}^\dagger\right)$
$x_{\rm zps}=p_{\rm zps}=\sqrt{\lambda/2}$ $\Rightarrow x_{\rm zps}p_{\rm zps}=\lambda/2$	$\varphi_{\rm zps}=2\pi\sqrt{\lambda}\frac{\Phi_{\rm zps}}{\Phi_0}$; $N_{\rm zps}=\sqrt{\lambda}\frac{Q_{\rm zps}}{2e}$ $\Rightarrow \varphi_{\rm zps}N_{\rm zps}=\lambda/2$
$[\hat{x},\hat{p}]=i\lambda$	$[\hat{\varphi},\hat{N}]=i\lambda$
$\hat{a}=\frac{1}{2}\left(\frac{\hat{x}}{x_{\rm zps}}+i\frac{\hat{p}}{p_{\rm zps}}\right)$	$\hat{a}=\frac{1}{2}\left(\frac{\hat{\varphi}}{\varphi_{\rm zps}}+i\frac{\hat{N}}{N_{\rm zps}}\right)$
$\hat{a}=(\hat{x}+i\hat{p})/\sqrt{2\lambda}$	$\hat{a}=\left(\sqrt{\frac{\lambda}{2}}\frac{\hat{\varphi}}{\varphi_{\rm zps}}+i\sqrt{\frac{\lambda}{2}}\frac{\hat{N}}{N_{\rm zps}}\right)/\sqrt{2\lambda}$

VI. THE RELATIONSHIP BETWEEN DIFFERENT SQUEEZE-DRIVEN KERR OSCILLATOR MODELS IN THE LITERATURE AND THEIR CLASSICAL LIMIT

In 1993, Wielinga and Milburn [13] proposed a quantum optical model that they called *the dynamical equivalent of the double-well potential.* The interest of the problem, to them, was that their model exhibited a double-well structure in phase space, and quantum mechanical ground state tunneling between them. The Hamiltonian they addressed is

$$\hat{H}_{\rm WM}=-K(\hat{a}^\dagger\hat{a})^2+\epsilon_2(\hat{a}^{\dagger 2}+\hat{a}^2). \tag{S1}$$

In 2017, the theoretical discovery of the Kerr-cat qubit by Puri, Boutin, and Blais [14] relied on the fact that the ground states of

$$\hat{H}_{\rm PBB}=-K\hat{a}^{\dagger 2}\hat{a}^2+\epsilon_2(\hat{a}^{\dagger 2}+\hat{a}^2) \tag{S2}$$

are fundamentally degenerate and exhibit no tunneling between two wells found in the classical limit (see also [15]). This property can be understood by writing Eq. (S2) into the factorized form [14]

$$\hat{H}_{\rm PBB}=-K(\hat{a}^{\dagger 2}-\epsilon_2/K)(\hat{a}^2-\epsilon_2/K), \tag{S3}$$

from which it is evident that the two coherent states $|\pm\alpha\rangle$ with $\alpha=\sqrt{\epsilon_2/K}$, which are the eigenstates of the annihilation operator $\hat{a}$, are also degenerate eigenstates of Eq. (S3). Since Eq. (S3) is negative-semidefinite and $\hat{H}_{\rm PBB}|\pm\alpha\rangle=0$, these states are the ground states.

Note that the Hamiltonians $\hat{H}_{\rm WM}$ and $\hat{H}_{\rm PBB}$ differ only by a commutator. Their shared classical limit can be written as

$$\begin{aligned} H_{\rm cl}&=-Ka^{*2}a^2+\epsilon_2(a^{*2}+a^2)\\ &=-K\left(\frac{x^2+p^2}{2}\right)^2+\epsilon_2(x^2-p^2). \end{aligned} \tag{S4}$$

Since $\hat{a}^{\dagger 2}\hat{a}^2 - (\hat{a}^\dagger\hat{a})^2 = \hat{a}^\dagger\hat{a}$, we cast the Hamiltonian

$$\hat{H} = \Delta\hat{a}^\dagger\hat{a} - K\hat{a}^{\dagger 2}\hat{a}^2 + \epsilon_2(\hat{a}^{\dagger 2} + \hat{a}^2), \tag{S5}$$

where we identify that $\hat{H}_{\text{PBB}}$ and $\hat{H}_{\text{WM}}$ are specific instances of Eq. (S5) with $\hat{H}_{\text{PBB}} = \hat{H}|_{\Delta=0}$ and $\hat{H}_{\text{WM}} = \hat{H}|_{\Delta=-K}$. Note that taking $\Delta \neq 0$ breaks the simple factorization condition of Eq. (S3). Indeed, the presence of the $\hat{a}^\dagger\hat{a}$ term is the cause of ground state tunneling in $\hat{H}_{\text{WM}}$, and its absence is the cause of the complete coherent cancellation of tunneling in $\hat{H}_{\text{PBB}}$. The lowest eigen-manifold of Eq. (S5) is plotted in Figure S5 while the excited state manifold of Eq. (S5) is plotted in Figure S6.

In 2007, Marthaler and Dykman [9] treated a Hamiltonian similar to Eq. (S5), where Δ was kept free for a fixed ϵ_2. This led to their prediction of periodic cancellation of tunneling amplitude for the ground state manifold as a function of Δ. Their work inspired our experiment shown in Figure 1 of the main text. We discuss in detail the mapping of their problem to ours in Section VIII.

In the following text, we discuss the quantum phase space representation of Eq. (S5).

VII. PHASE SPACE FORMULATIONS OF OUR EFFECTIVE HAMILTONIAN

Let us reconsider Eq. (S5). For its derivation starting from the circuit Hamiltonian, see appendix A of [2].

We obtain the phase space formulation of Eq. (S5) by taking the invertible Wigner transform [16] $\mathfrak{W}$ as

$$\begin{aligned}
&\hat{x} \to \mathfrak{W}\{\hat{x}\} = x; \qquad \hat{p} \to \mathfrak{W}\{\hat{p}\} = p;\\
&\hat{a} \to \mathfrak{W}\{\hat{a}\} = a = (x + ip)/\sqrt{2\lambda}; \quad \hat{a}^\dagger \to \mathfrak{W}\{\hat{a}^\dagger\} = a^*;\\
&\hat{a}^\dagger\hat{a} \to a^* \star a = a^*a - \frac{1}{2} = \frac{x^2+p^2}{2\lambda} - \frac{1}{2};\\
&\hat{a}^{\dagger 2}\hat{a}^2 \to a^{*2} \star a^2 = a^{*2}a^2 - 2a^*a + \frac{1}{2}\\
&\qquad\qquad\qquad = \frac{(x^2+p^2)^2}{4\lambda^2} - \frac{(x^2+p^2)}{\lambda} + \frac{1}{2};\\
&\hat{a}^{\dagger 2} + \hat{a}^2 \to a^{*2} + a^2 = \frac{(x^2-p^2)}{\lambda},
\end{aligned} \tag{S6}$$

where the Groenewold star product [17] is given by $\mathfrak{W}\{\hat{A}\hat{B}\} = A \star B = A \exp\left(\frac{1}{2}(\overleftarrow{\partial}_a \overrightarrow{\partial}_{a^*} - \overleftarrow{\partial}_{a^*} \overrightarrow{\partial}_a)\right) B$. The Moyal bracket [16, 18] over a and a^* is defined as $\{\!\{A, B\}\!\}_{a,a^*} = A \star B - B \star A$ so that we have $\{\!\{a, a^*\}\!\} = 1$. For a pedagogical exposition on the phase space formulation of quantum mechanics, we refer the reader to Section XI and [16, 19, 20]. With Eq. (S6), we write Eq. (S5) in the phase space formulation of quantum mechanics, up-to coordinate-independent terms, as

$$H = (\Delta + 2K)\left(\frac{x^2+p^2}{2\lambda}\right) - K\left(\frac{x^2+p^2}{2\lambda}\right)^2 + \epsilon_2\left(\frac{x^2-p^2}{\lambda}\right). \tag{S7}$$

Note that Eq. (S7) is not equal to Eq. (S4) even when $\Delta = 0$ and $\lambda = 1$. We further rescale Eq. (S7) by $-K/\lambda^2$ so as to have a coefficient of order 1 for the nonlinear term and rearrange Eq. (S7) as

$$\frac{-H\lambda^2}{K} = \left(\frac{x^2+p^2}{2}\right)^2 - \frac{2\epsilon_2\lambda}{K}\frac{x^2}{2}\left(1 + \frac{(\Delta+2K)}{2\epsilon_2}\right) + \frac{2\epsilon_2\lambda}{K}\frac{p^2}{2}\left(1 - \frac{(\Delta+2K)}{2\epsilon_2}\right). \tag{S8}$$

By choosing the scale of phase space $\lambda = K/2\epsilon_2$ Eq. (S8) becomes

$$\frac{-H\lambda^2}{K} = \left(\frac{x^2+p^2}{2}\right)^2 - \frac{x^2}{2}\left(1 + \frac{\Delta}{2\epsilon_2} + 2\lambda\right) + \frac{p^2}{2}\left(1 - \frac{\Delta}{2\epsilon_2} - 2\lambda\right). \tag{S9}$$

The term proportional to λ in Eq. (S9) involves a commutator, and corresponds to the Lamb shift. The classical limit then consist in dropping this term. This is valid for $\lambda \ll \min(\Delta/2\epsilon_2,\ 1)$. This translates to $\Delta/K,\ \epsilon_2/K \gg 1$. In this limit, the WKB approximation is valid to treat Eq. (1) in the main text.

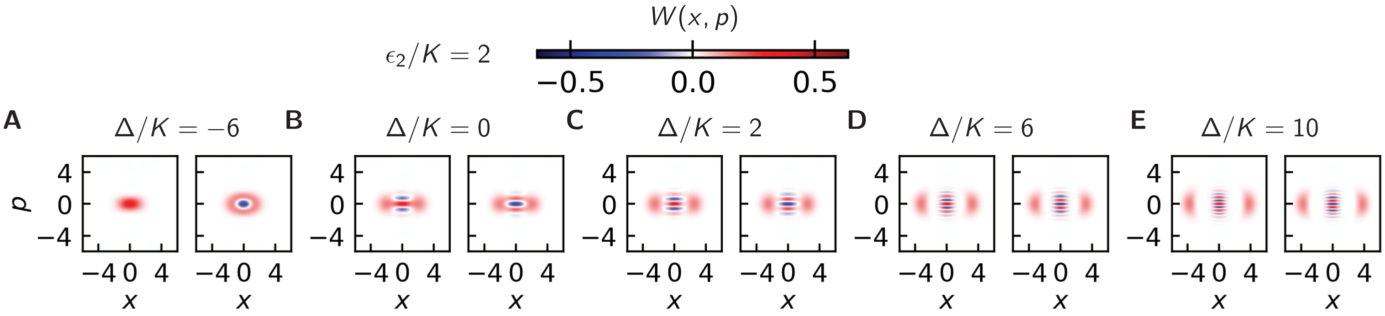

Figure S5: **Lowest eigen-manifold of the squeeze-driven Kerr oscillator** Wigner functions of lowest pair of eigenstates of Eq. (S5) (top row) for $\epsilon_2/K = 2$ and **A** $\Delta/K = -6$, **B** $\Delta/K = 0$, **C** $\Delta/K = 2$, and **D** $\Delta/K = 6$ and $\Delta/K = 10$ respectively. $\Delta/K \ll 0$, the eigenstates are squeezed. For $\Delta/K \ll 0$, increasing Δ/K yields Schrödinger cat states with increasing photon number. This phenomenon manifests as the monotonically growing baseline in transverse relaxation lifetime T_X in Figure 3 of the main text.

The interpretation of the Hamiltonian classical limit is that the elementary action element λ in the phase space defined by x and p must be much smaller than the typical dimensionless action of the system determined by the well-size parameters: Δ/K and ϵ_2/K. As we discuss in what follows (see section Section VII 1 and [2]), under the condition $\Delta/K,\ \epsilon_2/K \gg 1$, the wells of the Hamiltonian are large in the sense that they encompass many action quanta λ. Finally, note that for $\lambda \approx 1$ the classical treatment should not hold.

We call the surface for H in Eq. (S9) the metapotential of the squeeze-driven Kerr oscillator, and the classical limit for H in Eq. (S11) as the classical metapotential surface. Furthermore, as customary, we plot $-H$ rather than H to respect the familiar notion that in the presence of dissipation, stable equilibria correspond to well-bottoms rather than hill-tops.

1. *Properties of the metapotential surface*

In the table below, we examine the properties of the metapotential surface. For details on the number of levels inside the well, which we obtain via action quantization following the prescription of Einstein-Brillouin-Keller (EBK) [21], see Section VIII B.

Phase → ↓ Parameter (x, p phase space)	Double-node $-2\epsilon_2 \le \Delta + 2K \le 2\epsilon_2$	Triple-node $\Delta + 2K > 2\epsilon_2$
Area	$\frac{(\Delta+2K)}{K} \arccos\left(\frac{-(\Delta+2K)}{2\epsilon_2}\right) + \frac{2\epsilon_2}{K}\sqrt{1-\left(\frac{(\Delta+2K)}{2\epsilon_2}\right)^2}$	$\frac{4\epsilon_2}{K}\sqrt{\frac{(\Delta+2K)}{2\epsilon_2}-1} + \frac{2(\Delta+2K)}{K}\arcsin\left(\sqrt{\frac{2\epsilon_2}{(\Delta+2K)}}\right)$
Levels per well (#)	area$/2\pi - 1/2$	area$/2\pi - 1/2$
Approximation of #	$\frac{(\Delta+2K)/K}{2} + \frac{\epsilon_2/K}{\pi} - \frac{1}{2}$	$\frac{\sqrt{8\epsilon_2(\Delta+2K)}}{K\pi} - \frac{1}{2}$
Distance b/w nodes	$2\sqrt{\frac{(\Delta+2K)+2\epsilon_2}{K}}$	$2\sqrt{\frac{(\Delta+2K)+2\epsilon_2}{K}}$
Distance b/w saddles	0	$2\sqrt{\frac{(\Delta+2K)-2\epsilon_2}{K}}$
Depth of nodes	$\frac{(\Delta+2K+2\epsilon_2)^2}{4K}$	$\frac{(\Delta+2K+2\epsilon_2)^2}{4K}$
Depth of saddles	0	$\frac{(\Delta+2K-2\epsilon_2)^2}{4K}$
Depth of barrier	$\frac{(\Delta+2K+2\epsilon_2)^2}{4K}$	$\frac{2(\Delta+2K)\epsilon_2}{K}$

A. Wavefunctions of localized well states

In this section, we examine closely the wave functions of the squeeze-driven Kerr oscillator in the classically forbidden region and contrast them with those of an ordinary quadratic + quartic potential. We define the ordinary double-well

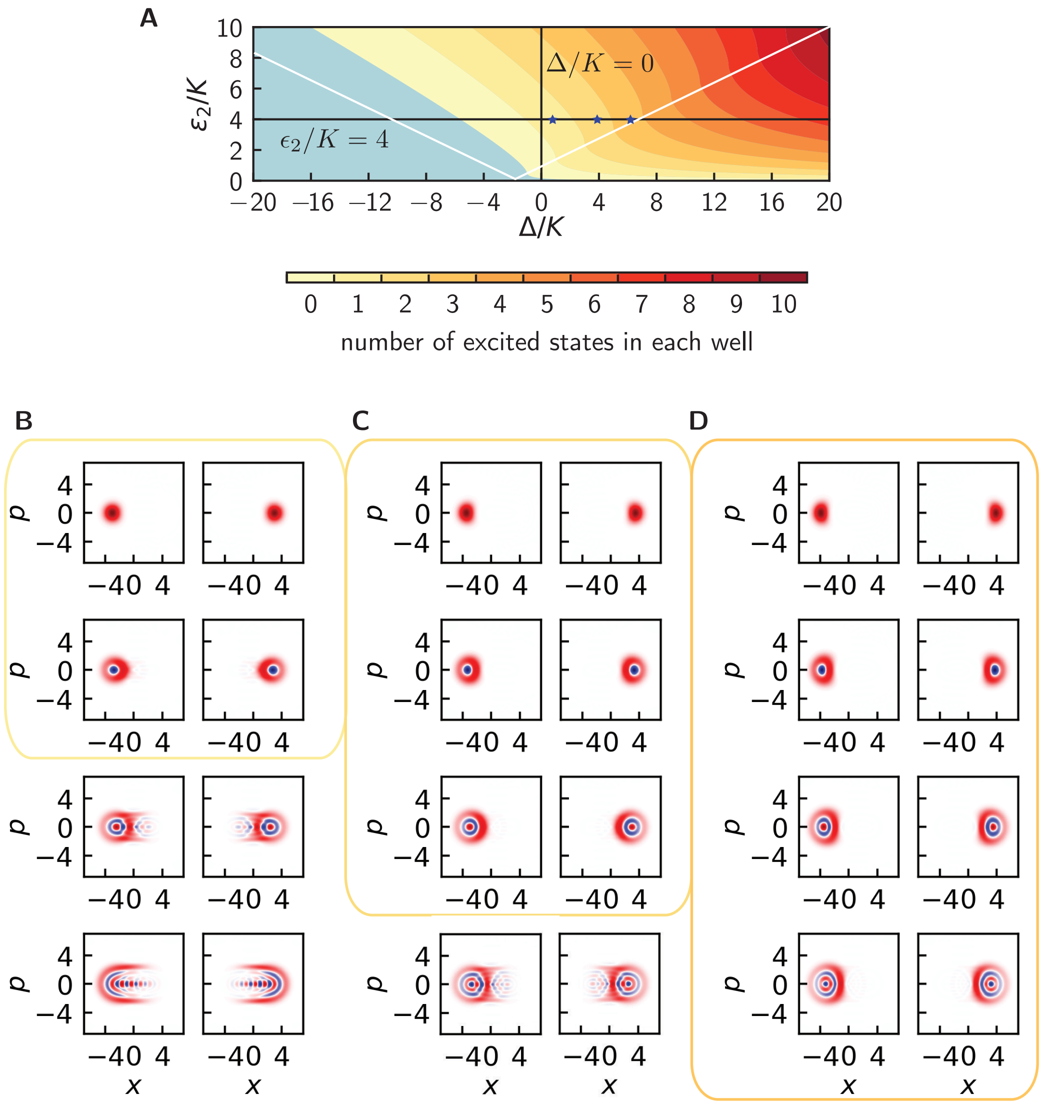

Figure S6: **Excited well states in the squeeze-driven Kerr oscillator** Localized ground and excited states in the squeeze-driven Kerr oscillator. **A** Period doubling phase diagram with equi-state contours. **B** - **E**. Wigner functions of exact eigenstates' superpositions, corresponding to localized states, for $\epsilon_2/K = 4$, and **B** $\Delta/K = 1$, **C** $\Delta/K = 4$, **D** $\Delta/K = 7$. The action quantization formulation, detailed in Section VIII and summarized by Eq. (S16), predicts **B** 1, **C** 2, **D** 3 excited states in each well. The Wigner functions of states outside this window are seen to have support in the other well too, and larger Δ helps localize them, thus validating the semiclassical picture discussed in Section VIII quantum mechanically.

Hamiltonian as

$$H = \frac{p^2}{2} + V(x), \qquad \text{with} \qquad V(x) = -\frac{k_2}{2}x^2 + \frac{k_4}{4}x^4,$$

where $k_2,\ k_4 > 0$. This potential has a saddle at $x_s = 0$, with $V(x_s) = 0$ and nodes at $x_n = \pm\sqrt{k_2/k_4}$ with the left and right well depth given by $V(x_n) = -k_2^2/(4k_4)$. The barrier height is given by $V(x_n) - V(x_s) = k_2^2/(4k_4)$.

The study of tunneling usually begins by considering a localized wave packet in one well, which is written as the superposition of the wavefunctions of the two lowest laying energy states ψ_+ and ψ_-.[2] Their energy difference is

---

[2] From a perturbation theory point of view this corresponds to the bonding and anti-bonding of the decoupled well states [22]. The zero point energy of the individual wells, in the absence of tunneling, is $E_0 = \sqrt{k_2}/2$. In the presence of tunneling the system's energies can be approximated by $E^{\pm} = E_0 \pm \delta E$.

denoted by $\delta E = E^+ - E^-$ and the left- and right-localized wavefunctions read

$$\psi_l = \frac{\psi_+ + \psi_-}{\sqrt{2}} \qquad \psi_r = \frac{\psi_+ - \psi_-}{\sqrt{2}}. \tag{S10}$$

On the left column of Figure S7, we plot the left and right-localized wavefunctions in red and blue respectively for **A** $k_2 = 3$, $k_4 = 1$, **B** $k_2 = 2$, $k_4 = 1$, and **B** $k_2 = 4$, $k_4 = 2$ respectively. The wavefunctions are computed by numerical diagonalization of the Hamiltonian. In the classically forbidden region, as one should expect, the wavefunctions display evanescent decay [23].

In the right column of Figure S7, we contrast the localized wavefunctions of the ordinary double-well potential with those of the squeeze-driven Kerr oscillator. The parameters Δ, K, and ϵ_2 were chosen so that a cut of the effective Hamiltonian surface at $p = 0$ yields an identical double-well potential as the left column. The wavefunctions of the full squeeze-driven Kerr oscillator are computed numerically. Importantly, **B**, **D**, and **F** show the localized wavefunctions for $\Delta/K = 0, 1$, and 2 respectively, corresponding to the destructive, constructive, and again destructive interference. Interestingly, in the classically forbidden region, in **B** and **D**, oscillations accompany decay in the wavefunction [9, 24]. This is due to the underlying driven nature of our system, providing a quartic term in momentum, which here reflects in the oscillatory nature of the wavefucntions in the classically forbidden region.

VIII. SEMICLASSICAL ANALYSIS

In [9], Marthaler and Dykman calculated the tunnel-splitting between states comprising the ground state manifold and found that the tunnel splitting vanishes periodically as a function of the drive frequency. We massage our phase-space Hamiltonian function into a form resembling Equation 5 of Marthaler and Dykman [9] by rewriting Eq. (S7) as

$$H = -\frac{K}{\lambda^2}\left[\left(\frac{x^2+p^2}{2}\right)^2 - \frac{x^2}{2}\left(1+\frac{\Delta}{2\epsilon_2}\right) + \frac{p^2}{2}\left(1-\frac{\Delta}{2\epsilon_2}\right)\right] \tag{S11}$$

where their parameter $\mu = (\Delta + 2K)/2\epsilon_2$.

A. WKB calculation of tunnel splitting for the ground state manifold of eq. (1)

The expression for the tunnel splitting following the analysis in [9, 24] is given as

$$\delta E = f \cos\theta \exp(-A), \tag{S12}$$

where

$$\begin{aligned}
f &= 2\left(\frac{4\epsilon_2}{K}\right)^2\left(\frac{K}{\pi(\Delta+2K)}\right)^{1/2}\left(1+\frac{(\Delta+2K)}{2\epsilon_2}\right)^{5/4}\\
\theta &= \frac{\pi}{2}\left(\frac{(\Delta+2K)}{K}-1\right)\\
A &= \frac{2\epsilon_2}{K}\left(\frac{(\Delta+2K)}{2\epsilon_2}+1\right)^{1/2} - \frac{(\Delta+2K)}{K}\log\left(\left(\frac{2\epsilon_2}{(\Delta+2K)}\right)^{1/2}+\left(1+\frac{2\epsilon_2}{(\Delta+2K)}\right)^{1/2}\right),
\end{aligned} \tag{S13}$$

where, the above expression is only valid for $(\Delta + 2K)/K \gg 1$. There are two failure modes for the WKB approximation. The first condition corresponds to when $\Delta \lesssim K$, and the other is when $\epsilon_2/K \ll 1$. Note that WKB works remarkably well outside its domain of validity ($\epsilon_2/K < 1$). Compare to Figure S7, where the wavelength given by the oscillation period of the wavefunction is of the same magnitude as the potential variation set by the interwell distance. Note that we have applied the formula developed in [9] in a domain that lies beyond the parameter regime where it was produced and we find remarkable agreement with data. The comparison between measured tunneling amplitude and a WKB theory can be found in Figure S8.

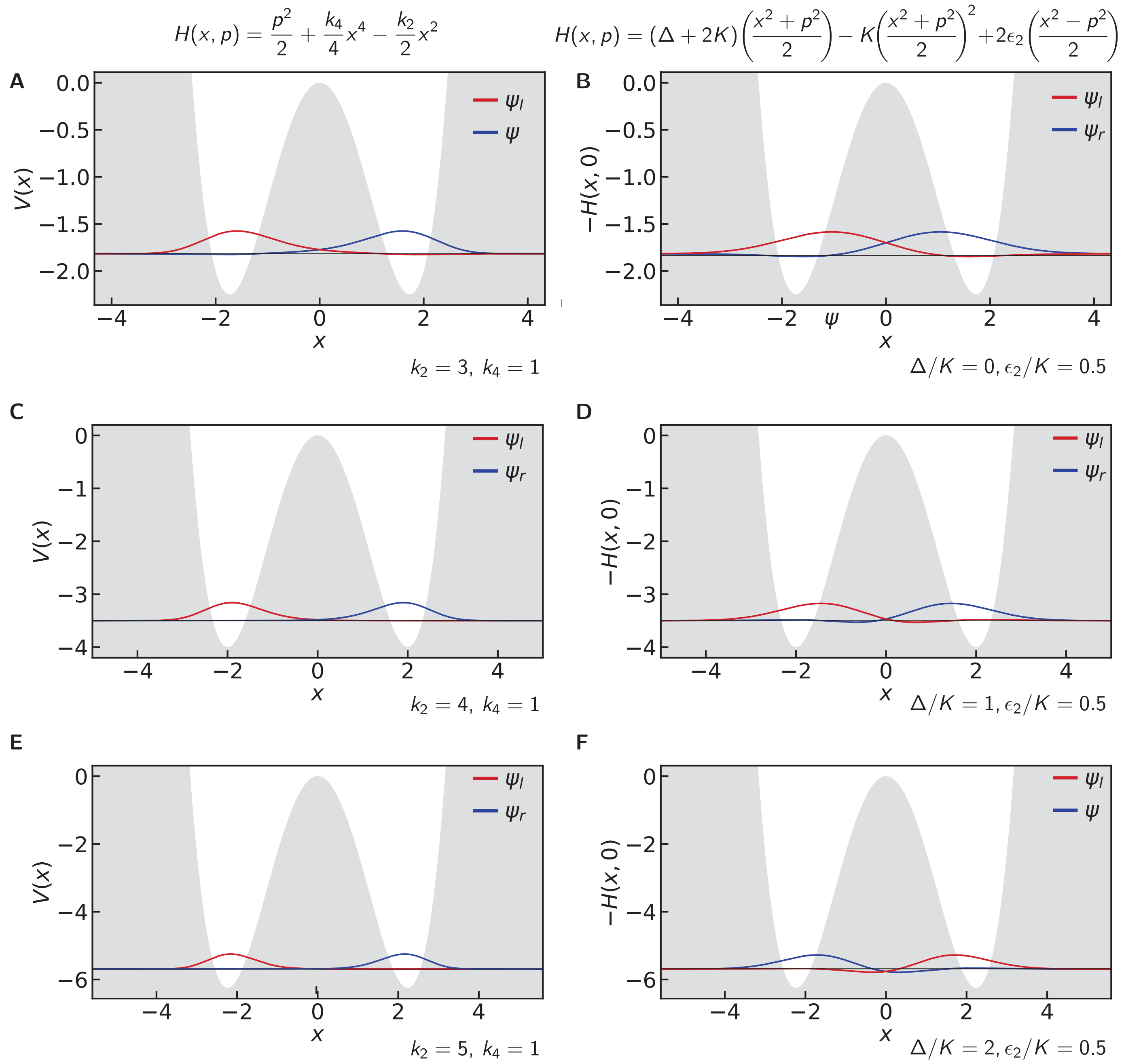

Figure S7: **Localized wavefunctions of the ground state manifold in the position basis in A, C, E an ordinary double well potential and in B, D, F for a squeeze-driven Kerr oscillator.** The Hamiltonian parameters in **A**, **C**, and **E** have been chosen to produce a double-well with the same depth and the well separation as those of **B**, **D**, and **F** respectively. The value of Δ/K is chosen to be **B** $\Delta/K = 0$, **D** $\Delta/K = 1$, and **F** $\Delta/K = 2$ corresponding to the destructive, constructive, and destructive interference of tunneling respectively. In the right panel, oscillations accompany decay of the wavefunction in the classically forbidden region, marked in grey. In the left panel, the wavefunction exhibits pure decay in the classically forbidden region. In this sense, the cancellation of tunneling amplitude in fig 1 of the main text can be understood as the destructive interference of the wavefunction in the classically forbidden region of the squeeze-driven Kerr oscillator. In [9], Marthaler and Dykman found an analytical expression for the WKB tunnel splitting of the ground state manifold. See Section VIII A for the WKB expressions for the tunnel splitting and Figure S8**B** and **D** for comparisons of the extracted tunnel splitting from experiment with their WKB theory.

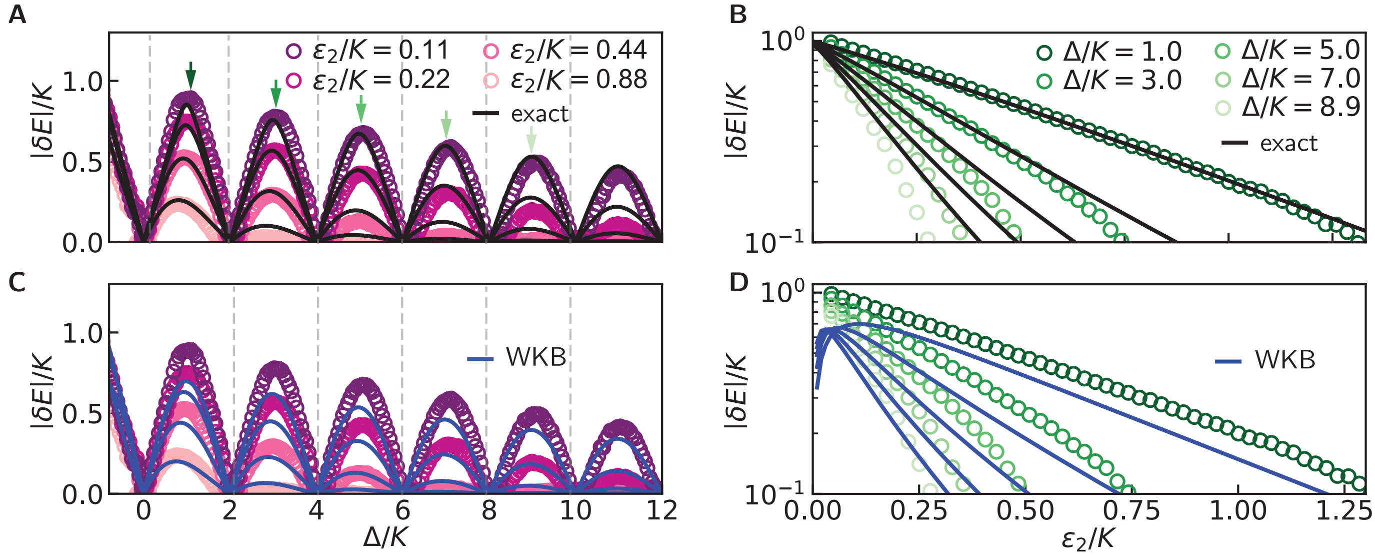

Figure S8: Experimentally extracted tunneling amplitude in the ground state manifold as a function of Δ (**A** and **C**) and ϵ_2 (**B** and **D**) compared to two different theoretical models. Dots correspond to extracted level splittings from dynamical measurements of the tunneling rate and correspond to the data presented in Figures 1C and 2 respectively. Solid lines in black in **A** and **B** are obtained via exact numerical diagonalization. Solid blue lines in **C** and **D** are obtained via a semi-classical WKB treatment developed by Marthaler and Dykman in [9, 24]. As expected, the semi-classical Hamiltonian model, in the domain of its validity $\Delta/K \gg 1$ and $\epsilon_2/K \sim 1$, agrees well with the measured data.

B. Action quantization via Einstein-Brillouin-Keller (EBK) method

In this section, we present the semiclassical method of obtaining the number of in-well states via action quantization, following the Einstein-Brillouin-Keller method, which generalizes the notion of Bohr orbits.

First, we introduce a polar-coordinate representation of Eq. (S11), which exploits its radial symmetry, as

$$H_{\rm cl} = \frac{\Delta r^2}{2} - \frac{K r^4}{4} + \epsilon_2 r^2 \cos 2\theta, \tag{S14}$$

where $x = r\cos\theta$ and $p = r\sin\theta$, for $r \geq 0$ and $\theta \in [0, 2\pi)$.

In a semiclassical treatment, a classical orbit $\mathcal{C}_j$ satisfying the following Einstein-Brillouin-Keller (EBK) quantization condition [21]:

$$\int_{\mathcal{C}_j} dx\, dp = \hbar \left(N_j + \frac{\beta_j}{4} \right), \tag{S15}$$

plays a special role. On the left hand side of Eq. (S15), the action integral corresponds to the area enclosed by the contour $\mathcal{C}_j$. On the right hand side of Eq. (S15), the non-negative integer $N_j \geq 0$ represents a quantum number and β_j is called a Maslov index; it counts the number of caustics encountered by the contour $\mathcal{C}_j$. For an orbit in the Kerr-cat metapotential, we have $\beta_j = 2$. Thus the condition in Equation (S15) states that only those orbits whose enclosed area satisfy a condition given by non-negative integers n_j and $\beta_j = 2$ correspond to allowed quantum orbits.

With this condition stated, one can ask a simple question: given a set of Δ, ϵ_2, how many in-well or bound states exist in the metapotential surface? This will be obtained by computing the number of allowed states at the separatrix, which separates bound and unbound states.

From the calculations detailed in Sections VIII B 1 and VIII B 2, we find the number of bound states as

$$N \sim \begin{cases} \frac{\Delta/K}{2} + \frac{\epsilon_2/K}{\pi} - \frac{1}{2} & -2\epsilon_2 \leq \Delta < 2\epsilon_2 \\ \frac{\sqrt{8\epsilon_2\Delta}}{K\pi} - \frac{1}{2} & \Delta \geq 2\epsilon_2. \end{cases} \tag{S16}$$

We demonstrate in Fig. S6 the value of the semi-classical action quantization condition in predicting the locality in phase space of even the excited states of the squeeze-driven Kerr oscillator.

1. *Separatrix area in the double-node phase:* $-2\epsilon_2 \leq \Delta < 2\epsilon_2$

In the double-node phase, the separatrix has a special name called the Bernoulli's lemniscate and its equation is given as

$$r^2 = \frac{2\Delta}{K} + \frac{4\epsilon_2}{K}\cos 2\theta, \tag{S17}$$

and $-\theta_c \leq \theta \leq \theta_c$, where $\theta_c = \frac{1}{2}\arccos\frac{-\Delta}{2\epsilon_2}$. We compute the area of a half the lemniscate as

$$\begin{aligned}
\int_{\mathcal{C}_j} dx\, p &= \frac{1}{2}\int_{-\theta_c}^{\theta_c} d\theta\, r^2 \\
&= \int_0^{\theta_c} d\theta\, \frac{2\Delta}{K} + \frac{4\epsilon_2}{K}\cos 2\theta \\
&= \frac{\Delta}{K}\arccos\left(\frac{-\Delta}{2\epsilon_2}\right) + \frac{2\epsilon_2}{K}\sqrt{1-\left(\frac{\Delta}{2\epsilon_2}\right)^2} \\
&\sim \frac{\Delta}{K}\left(\frac{\pi}{2} + \frac{\Delta}{2\epsilon_2}\right) + \frac{2\epsilon_2}{K}\left(1 - \frac{1}{2}\left(\frac{\Delta}{2\epsilon_2}\right)^2\right) \qquad |\Delta/2\epsilon_2| \ll 1 \\
&= \frac{\pi}{2}\frac{\Delta}{K} + 2\frac{\epsilon_2}{K}
\end{aligned} \tag{S18}$$

Note that for $\Delta = 0$, Eq. (S18) reduces to $2\epsilon_2/K$.

2. *Separatrix area in the triple-node phase:* $\Delta \geq 2\epsilon_2$

The separatrix in the triple-node phase is given as

$$r_\pm^2 = \frac{\Delta}{K} + \frac{2\epsilon_2}{K}\cos 2\theta \pm \frac{4\epsilon_2\cos\theta}{K}\sqrt{\frac{\Delta}{2\epsilon_2} - \sin^2\theta} \tag{S19}$$

and $-\theta_c \leq \theta \leq \theta_c$, where $\theta_c = \frac{\pi}{2}$. When plotted, this separatrix carves a bean-like shape.

Remarkably, we find an exact analytic expression for the area of this surface as

$$\begin{aligned}
\int_{\mathcal{C}_j} dx\, p &= \frac{1}{2}\int_{-\theta_c}^{\theta_c} d\theta\,(r_+^2 - r_-^2) \\
&= \int_{-\pi/2}^{\pi/2} d\theta\, \frac{4\epsilon_2\cos\theta}{K}\sqrt{\frac{\Delta}{2\epsilon_2} - \sin^2\theta} = \frac{4\epsilon_2}{K}\int_0^1 dt\sqrt{\frac{\Delta}{2\epsilon_2} - t^2} \\
&= \frac{4\epsilon_2}{K}\sqrt{\frac{\Delta}{2\epsilon_2} - 1} + \frac{2\Delta}{K}\arcsin\left(\sqrt{\frac{2\epsilon_2}{\Delta}}\right) \\
&\sim \frac{2\sqrt{8\epsilon_2\Delta}}{K}, \qquad \Delta/2\epsilon_2 \gg 1.
\end{aligned} \tag{S20}$$

IX. DEGENERACIES IN THE SQUEEZE-DRIVEN KERR OSCILLATOR

A. Robustness of degeneracies

The squeeze-driven Kerr oscillator we have engineered has the remarkable property: for $\Delta/K = 2m$, the first $m+1$ pairs of levels become decoupled from the rest of the oscillator's Hilbert space. Their eigenenergies and eigenstates become exactly solvable and present $m+1$ robust degeneracies in between states of different photon-number parity. Critically, note that the resonance condition for these degeneracies is independent of the value of the squeezing drive amplitude ϵ_2.

First, to show this, we begin by considering the squeezing drive as a perturbation to the Kerr oscillator described by the Hamiltonian $\hat{H}_K/\hbar = \Delta\hat{a}^\dagger\hat{a} - K\hat{a}^{\dagger 2}\hat{a}^2$ which is exactly solvable: its eigenstates are Fock states $|n\rangle$ and their energies are $E_n^{(0)} = \Delta n - Kn(n-1)$, which, as a function of Δ, are lines with integer slope that we plot in the top row of Figure S9**A**. The even(n)-odd(l) degeneracies read $E_n = E_l$ and imply $\Delta/K = 2m$ where $m = (n+l-1)/2 \geq 0$ is any nonnegative integer. In the second row, we plot the transition spectrum with respect to the ground state at $\epsilon_2 = 0$, which, due to the choice of rotating frame, corresponds to the *highest* energy eigenstate. This is the directly experimentally observable *transition* spectrum from the *ground* state. We further note that the ground state changes with Δ; remarkably, for $\epsilon_2 = 0$, at $\Delta/K = 2m$, the ground state is $(|m\rangle + |m+1\rangle)/\sqrt{2}$. This special property of the squeeze-driven Kerr oscillator has technological applications [25]. In the following rows, we plot the transition spectrum for increasing values of squeezing drive amplitude ϵ_2.

Indeed, it is clear that the squeezing drive renormalizes the energies of the Kerr oscillator. Level crossings of the Kerr oscillator with different parity remain exact crossings in the presence of the squeezing drive, since the interaction preserves parity. However, the remarkable feature is that these crossings are locked to where Δ equals an even multiple of K. In the following text, we justify this property, first via a perturbative and then provide a to-all-order proof.

1. *Perturbative analysis of degeneracies*

To first order in perturbation theory, we see that this even and odd Fock states remain decoupled (energy level crossings) under the parity conserving squeezing drive: $E_n^{(1)} = \langle n|(\hat{a}^{\dagger 2} + \hat{a}^2)|n+1\rangle = 0$. The condition for crossings of consecutive levels with different parity ($E_n = E_{n+2}$) reads instead $\Delta/K = (2n+1)$. To first order in perturbation theory, the avoided crossing amplitude is $E_n^{(1)} = \epsilon_2\sqrt{(n+1)(n+2)}$.

As a next approximation to the problem, we see the robustness of the crossings of consecutive levels with different parity (at $\Delta = 2nK$) by computing the second order correction to the nth energy levels $E_n^{(2)}$ and comparing it to the correction for the $(n+1)$th energy level

$$E_{n+1}^{(2)} = \epsilon_2^2\left(\frac{(n+3)(n+2)}{-2\Delta + 2K(2n+3)} + \frac{(n+1)n}{2\Delta - 2K(2n+1)}\right),$$

to find that $E_n^{(2)} = E_{n+1}^{(2)}$ for $\Delta/K = 2n$. This robustness can be seen in Figure S9**A** (all panels), where we see that the crossing shifts in energy but remains locked to Δ/K equal to even non-negative integers. The perturbation theory argument is easily generalized to non-consecutive level crossings and anti-crossings to this order. A similar perturbative argument was made in [25].

2. *Non-perturbative analysis of degeneracies*

To prove that the location of the degeneracies in Δ is independent of the squeezing drive amplitude to all orders we observe that we can write the Hamiltonian in Eq. (1) as

$$\hat{H} = \lambda_1(\hat{a}^{\dagger 2} - \alpha^2)(\hat{a}^2 - \alpha^2) + \lambda_2(\hat{a}^2 - \alpha^2)(\hat{a}^{\dagger 2} - \alpha^2), \tag{S21}$$

where, for $\Delta/K = 2m$ (m non-negative integer), we have $\lambda_1 = -K(1+m/2)$, $\lambda_2 = mK/2$ and $\alpha = \pm\sqrt{\epsilon_2/K}$, and which is a generalization of the factorization condition proposed in [14] for $\Delta = 0$. We next consider the displaced Hamiltonian $\hat{H}^+ = \hat{D}(+\alpha)\hat{H}\hat{D}^\dagger(+\alpha)$, which brings one of the wells to the origin of phase space. In this frame, the Hamiltonian operator can be written as[3]

$$\begin{aligned}\hat{H}^+ = &- K\left(\hat{a}^{\dagger 2}\hat{a}^2 + (4\alpha^2 + 2m)\hat{a}^\dagger\hat{a}\right)\\ &- 2K\alpha[\hat{a}^\dagger\hat{a} - (m+1)]\hat{a}^\dagger\\ &- 2K\alpha[\hat{a}^\dagger\hat{a} - m]\hat{a}.\end{aligned}$$

[3] Note that, without specializing Δ, one can directly write from Eq. (1) in the main text, or equivalently from Eq. (S21): $\hat{H}^+ = -K\left(\hat{a}^{\dagger 2}\hat{a}^2 + (4\alpha^2 + \Delta/K)\hat{a}^\dagger\hat{a}\right) - 2K\alpha[\hat{a}^\dagger\hat{a} - (\Delta/2K + 1)]\hat{a}^\dagger - 2K\alpha[\hat{a}^\dagger\hat{a} - \Delta/2K]\hat{a}$. From this expression one can directly derive the sub-space decoupling condition to be $\Delta/K = 2m$, in an exact manner, without relying in perturbative calculation or any previous knowledge existence of the resonance. The independence of the sub-space decoupling condition with respect to ϵ_2 is explicit.

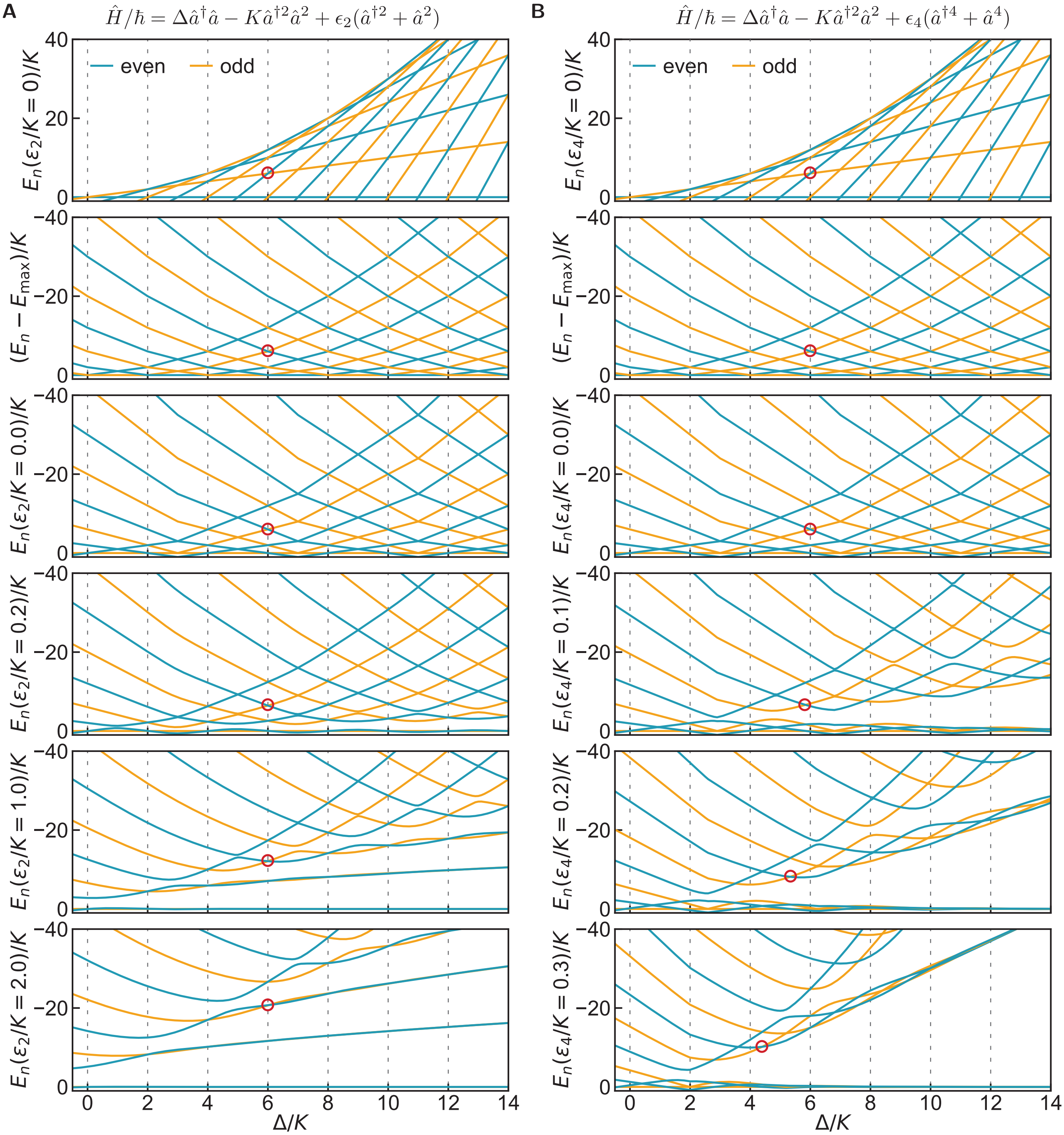

Figure S9: **Robustness of degeneracies in the squeeze-driven Kerr oscillator.** Spectrum of **A** Eq. (1) and **B** $\hat{H} = \Delta\hat{a}^\dagger\hat{a} - K\hat{a}^{\dagger 2}\hat{a}^2 + \epsilon_4(\hat{a}^{\dagger 4} + \hat{a}^4)$ as a function of Δ/K for different values of ϵ_2/K and ϵ_4/K respectively. Dashed lines mark Δ/K corresponding to even integers. Left panel indicates that even for non-perturbative values of ϵ_2/K, the locations of crossings of even (blue) and odd (orange) parity eigenstates occur at even values of Δ/K. Right panel indicates that even for the parity preserving perturbation controlled by ϵ_4/K, the locations of the crossings of even and odd parity states get renormalized. Red circle tracks one such crossing.

While the first line is number conserving, the next two lines couple only consecutive Fock states. In matrix form, it is tridiagonal in the Fock basis $|n\rangle$. By examining the square brackets in the above expression, we see that the off-diagonal elements are exactly zero for $n = m$ and $n = m + 1$. Thus, the first $m + 1$ states decouple from the rest of the oscillator's Hilbert space. The finite matrix is Hermitian, negative-semidefinite, and tridiagonal so it is exactly diagonalizable. Finally, we note that in phase space, a displacement of the metapotential surface, which is mirror-symmetric about $x = 0$, is identical to an opposite displacement composed with a rotation of 180° around the origin. Since the photon-number parity operator $\hat{\Pi} = e^{i\pi\hat{a}^\dagger\hat{a}}$ commutes with the Hamiltonian ($[\hat{\Pi}, \hat{H}] = 0$) the rotation is a symmetry of the system. Specifically; $\hat{H}^- = \hat{D}(-\alpha)\hat{H}\hat{D}^\dagger(-\alpha) \Rightarrow \hat{\Pi}\hat{H}^-\hat{\Pi} = \hat{H}^+$. We thus have two sets of equivalent[4] $m+1$ exactly solvable eigenenergies, and $2(m+1)$ linearly independent equations [5], which imply the existence of $m+1$ degeneracies in the spectrum for $\Delta/K = 2m$. The $2(m + 1)$ eigenstates $|\psi^\pm_{k\leq m+1}\rangle = \hat{D}(\pm\alpha)|\phi^\pm_{k\leq m+1}\rangle$ of $\hat{H}$, where $|\phi^-_{k\leq m+1}\rangle = \hat{\Pi}|\phi^+_{k\leq m+1}\rangle$ and $\hat{H}^+|\phi^+_{k\leq m+1}\rangle = E_{k\leq m+1}|\phi^+_{k\leq m+1}\rangle$, found in this way are not orthogonal, but thanks to the two-fold degeneracy condition we can take the superposition of the right (+) and left (−) kth displaced state to get an orthogonal basis in each of the $m+1$ two-fold degenerate sub-spaces: $|\mathcal{C}^\pm_{k\leq m+1}\rangle \propto D(+\alpha)|\phi^+_{k\leq m+1}\rangle \pm D(-\alpha)|\phi^-_{k\leq m+1}\rangle$. These $2(m + 1)$ pairwise-degenerate eigenstates of energy are also eigenstates of parity[6]. In this work we name these pairs of degenerate states the Δ-cats.

Note, that the robustness of the resonance condition is a peculiar symmetry property of the squeeze-driven Kerr oscillator and not a property of generic Kerr parametric oscillators. The existence of this robust degeneracies begs the question: what are the hidden symmetries associated with these degeneracies, if any? We show in Figure S9**B**, as an example, the spectrum of $\hat{H} = \Delta\hat{a}^\dagger\hat{a} - K\hat{a}^{\dagger 2}\hat{a}^2 + \epsilon_4(\hat{a}^{\dagger 4} + \hat{a}^4)$, where the location in Δ of the super-parity resonances depend on the value of the parametric drive amplitude ϵ_4. Note, also, that even if the multilevel resonances in Figure S9**B** are displaced with the value of the parametric drive amplitude (red circles), they are locked together to a running resonance condition: the point of exact solvability is changed by the drive. The phenomenon corresponds to deep symmetries [27, 28] of these type of, as of now, engineerable bosonic Hamiltonians and will be discussed in detail in a separate publication.

X. MODELING THE MEASURED TRANSVERSE RELAXATION LIFETIME T_X

To model the transverse relaxation lifetime measurements T_X of the Kerr-cat qubit, which we also refer to as the well-switching lifetime of the Kerr-cat system, we use a standard Lindblad master equation as:

$$\partial_t\hat{\rho} = \frac{1}{i\hbar}[\hat{H}, \hat{\rho}] + \kappa(1 + \bar{n}_{\text{th}})\mathcal{D}[\hat{a}]\hat{\rho} + \kappa\bar{n}_{\text{th}}\mathcal{D}[\hat{a}^\dagger]\hat{\rho}, \tag{S22}$$

where $\hat{\rho}$ describes the state of the system, $\bar{n}_{\text{th}} = 1/(\exp(\hbar\omega_a/k_BT) - 1)$ corresponds to the temperature of the environment and κ corresponds to the coupling between system and environment. The Hamiltonian $\hat{H}$ is given by Eq. (S5) and the dissipator $\mathcal{D}$ of the operator $\hat{O}$ is given by $\mathcal{D}[\hat{O}]\bullet := \hat{O}\bullet\hat{O}^\dagger - (\hat{O}^\dagger\hat{O}\bullet + \bullet\hat{O}^\dagger\hat{O})/2$. In Eq. (S22), these operators correspond to single photon loss $\mathcal{D}[\hat{a}]$ and gain $\mathcal{D}[\hat{a}^\dagger]$ [29–31]. In Figure S10, we compare the data presented in Figure 3 of the main text with the lifetime extracted from Eq. (S22) for different values of n_{th}. The value of κ has been set to $\kappa = 1/T_1 = 1/20$ µs^{-1}. The current model seems insufficient to accurately predict the observations and more research is needed to understand the decoherence of nonlinear driven systems (see, for example, [32]). Figure S10 emphasizes the need for further measurements and a detailed modeling of possible noise sources

---

[4] Note that for the off-diagonal elements the parity transformation produces a minus sign ($\hat{\Pi}|n\rangle\langle n \pm 1|\hat{\Pi} = -|n\rangle\langle n \pm 1|$) that manifests in $\alpha \to -\alpha$: $H^+_{n,n\pm1} = -H^-_{n,n\pm1}$. This leaves the finite characteristic polynomial invariant.

[5] The elements of the finite set of eigenvectors of $\hat{H}^+$, $\{|\phi^+_{k\leq m+1}\rangle\}_k$, are linearly independent from the finite set of eigenvectors of $\hat{H}^-$, $\{|\phi^-_{k\leq m+1}\rangle\}_k$, since they are spanned by the first $m + 1$ displaced Fock states in different directions (±, see text). Indeed, any Fock state i that is displaced has support in all (undisplaced) Fock states j's [26]: for $j > i$ the formula reads $|\langle j|\hat{D}(\pm 2\alpha)|i\rangle| = \left(\frac{i!}{j!}\right)^{1/2}|2\alpha|^{j-i}e^{-2|\alpha|^2}|L_i^{(j-i)}(4|\alpha|^2)| > 0$, where $L_i^{(j-i)}$ is an associated Laguerre polynomial (note that the matrix element tends to zero rapidly as $|\alpha|$, or equivalently $|\epsilon_2|$, grows, while the decoupled subspace condition, and ultimately the proof itself, is independent of these values for as long as they are non zero. If $\epsilon_2 = 0$, the proof is trivial and is given in the previous subsection). In other words, the linear independence manifests here explicitly in that $|\phi^\pm_{k\leq m+1}\rangle$ have no defined parity, yet $|\phi^+_{k\leq m+1}\rangle = \hat{\Pi}|\phi^-_{k\leq m+1}\rangle$ (note that $[\hat{\Pi}, \hat{H}^\pm] \neq 0$). Ultimately, $|\langle\phi^-_{k\leq m+1}|\hat{D}^\dagger(-\alpha)\hat{D}(+\alpha)|\phi^+_{k\leq m+1}\rangle| = |\langle\phi^+_{k\leq m+1}|\hat{D}(2\alpha)|\phi^+_{k\leq m+1}\rangle| < 1$ if $|\alpha| > 0$.

[6] Specifically $\hat{\Pi}|\mathcal{C}^\pm_{k\leq m+1}\rangle = \pm|\mathcal{C}^\pm_{k\leq m+1}\rangle$ and are thus orthogonal. We used $\hat{D}(+\alpha)\hat{\Pi} = \hat{\Pi}\hat{D}(-\alpha)$.

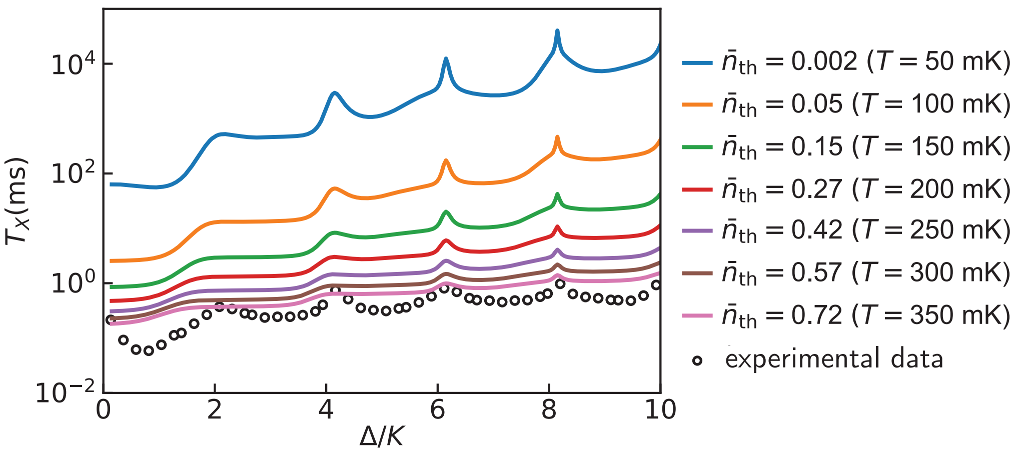

Figure S10: **Lindblad simulations of T_X as a function of Δ for different thermal populations, corresponding to Eq. (S22).** Black dots correspond to experimental data presented in Fig. 3 **G** in the main text. The value of κ has been taken as $\kappa = 1/T_1 = 1/20\ \mu s^{-1}$ and the value of ϵ_2 has been chosen as $\epsilon_2/K = 2.17$ to match the experimental data. The solid curves take the experimentally observed ac Stark shift into account. An ordinary Lindbladian at non-zero temperature is insufficient to predict the experimental data. Beyond-RWA effects may be important to consider [32]. See also [33].

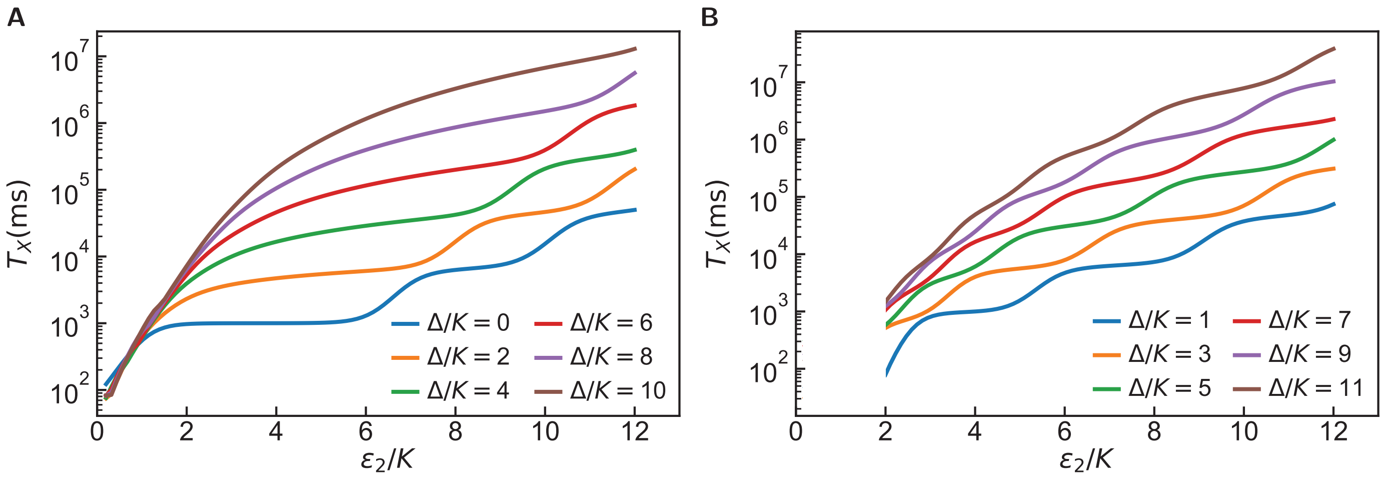

Figure S11: **Ordinary Lindblad simulations of T_X as a function of ϵ_2/K for different values of Δ/K,, corresponding to Eq. (S22).** For both **A** and **B**, the value of $\kappa/K = 1/50$ and $\bar{n}_{th} = 0.05$. In **B** for $\epsilon_2/K < 2$ the lifetime is limited by ground state tunneling and is this not well captured by our simplified method.

affecting particularly driven qubits. See also the note at the end of Section XI. We also present, in Figure S11, the expected T_X as a function of ϵ_2/K for different values of Δ. This plot indicates that a Δ-Kerr-cat, in general, gives larger T_X lifetimes than a Kerr-cat ($\Delta = 0$).

XI. TUTORIAL ON THE PHASE SPACE FORMULATION OF QUANTUM MECHANICS

A full quantum mechanical treatment can be developed in phase space without incurring in any semiclassical approximations [16–18]. For the sake of completeness, here we provide an overview on the mapping from operator-valued Hilbert space to quantum phase space and a few elemental techniques and identities. We focus here on Wigner phase space, and showcase that the Wigner transform is more than a visualization tool for states. We note that our treatment can be equivalently extended to other phase space formulations [16, 19, 34–36].

From operator Hilbert space to Wigner phase space (and back)

The Wigner transform [37] of the density matrix $\hat{\rho}$ is the Wigner function $W(X, P)$, where X and P are standard phase space coordinates (not operators) with dimensions of position and momentum (see Section V for notation). We write this as

$$\mathfrak{W}\{\hat{\rho}\} = W(X, P).$$

Let us remind the reader of some crucial properties of the Wigner function. We have

$$\iint dXdP\, W(X, P) = 1, \tag{S23}$$

where each integral runs from $-\infty$ to ∞ and we suppress the limits in the following text for simplicity. For a pure state, we further have

$$h \iint dXdP\, W(X, P)^2 = 1, \tag{S24}$$

where $h = 2\pi\hbar$.

In general, we have

$$0 \leq h^{n-1} \iint dXdP\, W(X, P)^n \leq 1, \tag{S25}$$

which corresponds to the positivity of the density matrix.

Likewise, for a generic operator $\hat{F}$, we introduce the phase space function $F(X, P) = \mathfrak{W}\{\hat{F}\}$.

In this framework, the average value of an Hermitian operator $\hat{F}$ can be written as

$$\langle \hat{F} \rangle = \iint dXdP\, F(X, P)W(X, P). \tag{S26}$$

The transformation $\mathfrak{W}$ is invertible as appreciated by Groenewold [17]

$$\mathfrak{W}^{-1}\{W(X, P)\} = \hat{\rho}.$$

The inverse transformation $\mathfrak{W}^{-1}$ is know as the Weyl transformation [38].

In general, the Weyl transformation is

$$\hat{\rho} = \mathfrak{W}^{-1}\{W\} = \frac{1}{h} \iiiint dXdPdkdl\, W(X, P) e^{\frac{i}{\hbar}(k(\hat{X}-X)+l(\hat{P}-P))}, \tag{S27}$$

where the characteristic function $C(l, k)$ defined as

$$C(l, k) = \iint dXdP\, e^{-\frac{i}{\hbar}(kX+lP)} W(X, P), \tag{S28}$$

is the Fourier transform of the Wigner function and C is dimensionless.

Another useful formula is

$$W(X, P) = \frac{1}{h} \int dq\, e^{-iqP/\hbar} \langle X + q/2|\hat{\rho}|X - q/2\rangle, \tag{S29}$$

where $\hat{\rho}$ is to be understood in the continuous position basis and therefore has the dimension of [1/position].

We now review simple operational rules to go from operator space to phase space functions and back without performing cumbersome integrals.

The Wigner and Weyl transformation take a particularly simple form for binomial expansions

$$\mathfrak{W}\{(\alpha\hat{X}+\beta\hat{P})^n\} = (\alpha X+\beta P)^n.$$

$$\mathfrak{W}^{-1}\{(\alpha X+\beta P)^n\} = (\alpha\hat{X}+\beta\hat{P})^n.$$

For non-symmetric expressions, the Wigner transform can be evaluated via a non-commutative Wigner phase space product, the celebrated Groenwold's star product.

A. An introduction to the star product

We introduce the star product as

$$\mathfrak{W}(\hat{F}\hat{G}) = \mathfrak{W}(\hat{F})\star\mathfrak{W}(\hat{G}) = F(X,P)\star G(X,P), \tag{S30}$$

defined as (the exponential of the Poisson bracket):

$$\begin{aligned} F\star G &= \sum_{n=0}^{\infty}\sum_{k=0}^{n}\frac{(-1)^k}{n!}\left(\frac{i\hbar}{2}\right)^n\binom{n}{k}\partial_P^k\partial_X^{n-k}F\times\partial_P^{n-k}\partial_X^k G \\ &\equiv F\exp\left(\frac{i\hbar}{2}\left(\overleftarrow{\partial}_X\overrightarrow{\partial}_P-\overleftarrow{\partial}_P\overrightarrow{\partial}_X\right)\right)G \\ &= FG+\frac{i\hbar}{2}\{F,G\}+\cdots \end{aligned} \tag{S31}$$

Here $F\overleftarrow{\partial}_X G = (\partial_X F)G$ and $F\overrightarrow{\partial}_X G = F(\partial_X G)$, and we have introduced the Poisson bracket $\{F,G\} = \partial_X F\partial_P G - \partial_P F\partial_X G$. The star product can also be conveniently expressed in terms of complex-coordinates a and a^* as

$$F\star G \equiv F\exp\left(-\frac{1}{2}\left(\overleftarrow{\partial}_{a^*}\overrightarrow{\partial}_a-\overleftarrow{\partial}_a\overrightarrow{\partial}_{a^*}\right)\right)G.$$

It generalizes to a system of many particles (or many modes) as

$$F\star G = F\exp\left(\frac{i\hbar}{2}\sum_j\left(\overleftarrow{\partial}_{X_j}\overrightarrow{\partial}_{P_j}-\overleftarrow{\partial}_{P_j}\overrightarrow{\partial}_{X_j}\right)\right)G.$$

In Fourier space the star product becomes a phase factor: $\star\rightarrow e^{i\frac{\hbar}{2}(k_X k'_P - k'_X k_P)}$ [39]. This phase corresponds to an oriented area in reciprocal phase space. This is the simplest manifestation of the noncommutativity of the algebra of quantum mechanics in phase space.

Remarkably, the scalar product associated with the star product is the usual integral in phase space. For phase space functions in the Wigner representation F and G, we have

$$\iint dXdP\,F(X,P)\star G(X,P) = \iint dXdP\,F(X,P)G(X,P). \tag{S32}$$

Note however that in general for any $F(X,P)$, $G(X,P)$, and $H(X,P)$,

$$\iint dXdP\,F(X,P)\star G(X,P)\star H(X,P) \neq \iint dXdP\,F(X,P)G(X,P)H(X,P). \tag{S33}$$

For non-symmetric expressions in $\hat{X}$ and $\hat{P}$, the above formulae can be employed to evaluate the Wigner transform. For example

$$\mathfrak{W}\{\hat{X}\hat{P}\hat{P}\} = XP^2 + i\hbar P$$

$$\mathfrak{W}\{\hat{P}\hat{P}\hat{X}\} = XP^2 - i\hbar P$$

$$\mathfrak{W}\{\hat{P}\hat{X}\hat{P}\} = XP^2.$$

We evaluate the Weyl transform of asymmetric expressions by symmetrizing it and replacing phase space functions by their corresponding operators. For example

$$\mathfrak{W}^{-1}\{XP^2\} = \frac{1}{3}(\hat{X}\hat{P}\hat{P} + \hat{P}\hat{X}\hat{P} + \hat{P}\hat{P}\hat{X}).$$

To find the Weyl transform of a high-degree polynomial of X and P, the Weyl-symmetrized form might be too tedious and McCoy [40] provided a shortcut to obtain polynomial expressions in the phase space representation. We review McCoy's formula in the next section.

The McCoy formula for obtaining ordered operators from phase space functions

While a fully-symmetrized representation is usually inconvenient for polynomials of large degree, McCoy derived a set of formulae [40], each corresponding to a different representation of a Weyl transform. Here, we present two of them that yield operators that privilege the ordering of $\hat{X}$ (or $\hat{P}$).

Consider a phase space function $F(X, P)$. Its operator-valued correspondent $\hat{F}$ in normal order with respect to X is given by the McCoy formula [40] that reads:

$$\mathcal{F}(X, P) = e^{-i\frac{\hbar}{2}\partial_X\partial_P} F(X, P)$$

$$\mathcal{F}(X, P) = F(X, P) - \frac{i\hbar}{2}\partial_X\partial_P F(X, P) - \frac{1}{2!}\frac{\hbar^2}{2^2}\partial_X^2\partial_P^2 F(X, P) + \cdots$$

$$\hat{F} = (\mathcal{N}_X\mathcal{F})|_{(\hat{X},\hat{P})},$$

The functional (operator over real functions) $\mathcal{N}_X$ is carried out by writing its arguments with X factors (or P factors as indicated by the subindex of $\mathcal{N}$) to the left in each term and replace X, P with $\hat{X}, \hat{P}$ respectively.

For example, if $F = XP$, we have $\mathcal{F} = XP - i\frac{\hbar}{2}$ which gives the correct and now ordered Hermitian expression for the operator $\hat{F} = \hat{X}\hat{P} - i\frac{\hbar}{2} = \frac{\hat{X}\hat{P}+\hat{P}\hat{X}}{2}$.

The inverse transform, is simply given $F(X, P) = e^{i\frac{\hbar}{2}\partial_X\partial_P}\mathcal{F}(X, P)$.

In terms of complex coordinates, $a = \frac{1}{\sqrt{2}}(x + ip)$ we adapt McCoy's formula [40]:

$$\mathcal{F}(a, a^*) = e^{\frac{1}{2}\partial_a\partial_{a^*}} F(a, a^*)$$

$$\hat{F} = (\mathcal{N}_{a^*}\mathcal{F})|_{(\hat{a},\hat{a}^\dagger)}$$

to get the normal ordered (with respect to a^*) result. For example, one has classically that $\frac{1}{2}(x^2 + p^2) = a^*a$. The correct quantization reads $F = aa^* \to \mathcal{F} = aa^* + 1/2 \to \hat{F} = \hat{a}^\dagger\hat{a} + 1/2$.

Application to our Hamiltonian

If the Wigner phase space Kerr Hamiltonian reads $H = \Delta a^* a - K a^{*2} a^2$ the corresponding operator is

$$F = a^{*2} a^2 \to \mathcal{F} = a^{*2} a^2 + 2a^* a + \frac{1}{2} \to \hat{F} = \hat{a}^{\dagger 2} \hat{a}^2 + 2\hat{a}^\dagger \hat{a} + \frac{1}{2},$$

$$\hat{H}/\hbar = (\Delta - 2K)\hat{a}^\dagger \hat{a} - K\hat{a}^{\dagger 2}\hat{a}^2,$$

where the oscillator frequency is renormalized by $2K$. This is the Lamb shift, and its origin is in the non commutativity of $\hat{a}$ and $\hat{a}^\dagger$, i.e. the vacuum fluctuations.

Groenewold's theorem

Note that $\mathfrak{W}\left\{\frac{1}{i\hbar}[\hat{F}, \hat{G}]\right\} = \{\!\{\mathfrak{W}(\hat{F}), \mathfrak{W}(\hat{G})\}\!\} \neq \{\mathfrak{W}(\hat{F}), \mathfrak{W}(\hat{G})\}$. The quantum commutators do not correspond to the Poisson brackets: the theorem [17] states that such a mapping does not exist. We provide a practical consequence of the implications of this theorem to quantum Hamiltonian engineering in Appendix B of [41].

Dynamics of the Wigner function: the Moyal equation

The von-Neumann equation $\partial_t \hat{\rho} = \frac{1}{i\hbar}[\hat{H}, \hat{\rho}]$ (the density-operator version of the Schrödinger equation) transforms as

$$\partial_t W = \frac{1}{i\hbar}(H \star W - W \star H),$$

$$\partial_t W = \{\!\{H, W\}\!\}.$$

Here $H(X, P) = \mathfrak{W}(\hat{H})$ is the Hamiltonian function and we have introduced the Moyal bracket notation [18]. We refer the reader to [20] for a derivation of the equation of motion of the Wigner function from Schödinger's equation for the wavefunction without referring to the star product.

The exponential notation of the star product induces the name "Moyal sine bracket" since it can be written as

$$\partial_t W = H \frac{2}{\hbar} \sin\left(\frac{\hbar}{2}\left(\overleftarrow{\partial}_X \overrightarrow{\partial}_P - \overleftarrow{\partial}_P \overrightarrow{\partial}_X\right)\right) W.$$

Note that the Moyal equation is identical to Liouville equation plus quantum corrections coming from the expansion of the sine to higher orders of $\hbar$.

$$\partial_t W = \{H, W\} + \mathcal{O}(\hbar^2).$$

Interestingly, there is no corrections to $\mathcal{O}(\hbar)$. Importantly, the quantum corrections are proportional to $\hbar^2$ and to the nonlinear terms in the Hamiltonian. For quadratic Hamiltonians, all the quantum corrections vanish: the higher-order derivatives exterminate low-order polynomials (see the Appendix of [2]). Specifically, Gaussian transformations, i.e., those generated by quadratic Hamiltonians in the phase space coordinates, are classical in the sense that they are ruled by only the Poisson bracket. Thus, they would not develop negativities in the Wigner distribution if none would be present at the beginning.

Phase space formulation for open quantum systems

So far, we have only discussed the phase space formulation for closed quantum systems. Indeed, one can extend the treatment to open systems as we demonstrate below. The Lindblad equation for single photon loss is given by

$$\partial_t\hat{\rho} = \frac{1}{i\hbar}[\hat{H},\hat{\rho}] + \kappa\hat{a}\hat{\rho}\hat{a}^\dagger - \frac{\kappa}{2}(\hat{a}^\dagger\hat{a}\hat{\rho} + \hat{\rho}\hat{a}^\dagger\hat{a}). \tag{S34}$$

Using Eq. (S30) and Eq. (S31), we get the phase space formulation of Eq. (S34) as

$$\mathfrak{W}\{\partial_t\hat{\rho}\} = \partial_t W,$$

$$\begin{aligned}\mathfrak{W}\left\{\frac{1}{i\hbar}[\hat{H},\hat{\rho}]\right\} &= \{\!\{\mathfrak{W}(\hat{H}),\mathfrak{W}(\hat{\rho})\}\!\} \\ &= \{\!\{H,W\}\!\}\end{aligned}$$

$$\begin{aligned}\mathfrak{W}\{\hat{a}\hat{\rho}\hat{a}^\dagger\} &= a\star W\star a^* \\ &= aWa^* + \frac{1}{2}\partial_a W + \frac{1}{2}(\partial_{a^*}(Wa^*) + \frac{1}{2}\partial^2_{aa^*}W)\end{aligned}$$

$$\begin{aligned}\mathfrak{W}\{\hat{a}^\dagger\hat{a}\hat{\rho}\} &= \left(a^*a - \frac{1}{2}\right)\star W \\ &= aWa^* - \frac{1}{2}W + \frac{1}{2}(a^*\partial_{a^*}W - a\partial_a W) - \frac{1}{4}\partial^2_{aa^*}W\end{aligned}$$

$$\begin{aligned}\mathfrak{W}\{\hat{\rho}\hat{a}^\dagger\hat{a}\} &= W\star\left(a^*a - \frac{1}{2}\right) \\ &= aWa^* - \frac{1}{2}W - \frac{1}{2}(a^*\partial_{a^*}W - a\partial_a W) - \frac{1}{4}\partial^2_{aa^*}W\end{aligned}$$

Gathering all terms one directly gets

$$\partial_t W = \{\!\{H,W\}\!\} + \frac{\kappa}{2}\left(\partial^2_{aa^*} + \partial_a a + \partial_{a^*}a^*\right)W.$$

It is convenient to translate the above to x,p space

$$\partial_t W = \{\!\{H,W\}\!\} + \frac{\kappa}{2}\left(\partial^2_x + \partial^2_p + \partial_x x + \partial_p p\right)W. \tag{S35}$$

By expressing the equation in x,p space in Eq. (S35), the diffusion terms $\propto(\partial^2_x+\partial^2_p)$ and the drag terms $\propto(\partial_x x+\partial_p p)$ associated to the fluctuation and the dissipation become evident. Note, that the Moyal sine bracket has only odd derivatives: the diffusion $(\partial^2_x+\partial^2_p)$ cannot be canceled by Hamiltonian dynamics.

For finite temperature $\bar{n}_{\text{th}}$, the Lindblad master equation is

$$\partial_t\hat{\rho} = \frac{1}{i\hbar}[\hat{H},\hat{\rho}] + \kappa(1+\bar{n}_{\text{th}})\mathcal{D}[\hat{a}]\hat{\rho} + \kappa\bar{n}_{\text{th}}\mathcal{D}[\hat{a}^\dagger]\hat{\rho}, \tag{S36}$$

where the dissipator $\mathcal{D}$ of the operator $\hat{O}$ is given by $\mathcal{D}[\hat{O}]\bullet := \hat{O}\bullet\hat{O}^\dagger - (\hat{O}^\dagger\hat{O}\bullet + \bullet\hat{O}^\dagger\hat{O})/2$.

It is straightforward to show that in the phase space formulation, Eq. (S36) reads

$$\partial_t W = \{\!\{H,W\}\!\} + \frac{\kappa}{2}\left(\partial_a a + \partial_{a^*}a^*\right)W + \kappa\left(\frac{1}{2}+\bar{n}_{\text{th}}\right)\partial^2_{a^*a}W, \tag{S37}$$

which reads in x,p space as

$$\partial_t W = \{\!\{H,W\}\!\} + \frac{\kappa}{2}\left(\partial_x x + \partial_p p\right)W + \frac{\kappa}{2}\left(\frac{1}{2}+\bar{n}_{\text{th}}\right)\left(\partial^2_x + \partial^2_p\right)W. \tag{S38}$$

Equation (S38) is the quantum version of the Fokker-Planck equation, with the Poisson bracket replaced by the Moyal bracket and a quantum diffusion term corresponding to the zero point spread.

Note that for the Hamiltonian corresponding to Eq. (S5), the solution for W from Eq. (S38) will not yield the Boltzmann distribution in steady state, which perhaps is not surprising for an out-of-equilibrium driven problem [42].

---

[1] A. Grimm, N. E. Frattini, S. Puri, S. O. Mundhada, S. Touzard, M. Mirrahimi, S. M. Girvin, S. Shankar, and M. H. Devoret, Nature **584**, 205 (2020).
[2] N. E. Frattini, R. G. Cortiñas, J. Venkatraman, X. Xiao, Q. Su, C. U. Lei, B. J. Chapman, V. R. Joshi, S. Girvin, R. J. Schoelkopf, et al., arXiv preprint arXiv:2209.03934 (2022).
[3] A. Blais, A. L. Grimsmo, S. M. Girvin, and A. Wallraff, Reviews of Modern Physics **93**, 025005 (2021).
[4] D. Roberts and A. A. Clerk, Physical Review X **10**, 021022 (2020).
[5] D. Sank, Z. Chen, M. Khezri, J. Kelly, R. Barends, B. Campbell, Y. Chen, B. Chiaro, A. Dunsworth, A. Fowler, et al., Physical review letters **117**, 190503 (2016).
[6] R. Shillito, A. Petrescu, J. Cohen, J. Beall, M. Hauru, M. Ganahl, A. G. M. Lewis, G. Vidal, and A. Blais, Dynamics of transmon ionization (2022).
[7] J. Cohen, A. Petrescu, R. Shillito, and A. Blais, Reminiscence of classical chaos in driven transmons (2022).
[8] A. Petrescu, M. Malekakhlagh, and H. E. Türeci, Physical Review B **101**, 134510 (2020).
[9] M. Marthaler and M. Dykman, Physical Review A **76**, 010102 (2007).
[10] M. H. Devoret et al., Les Houches, Session LXIII **7**, 133 (1995).
[11] S. M. Girvin, Quantum machines: measurement and control of engineered quantum systems pp. 113–256 (2014).
[12] M. H. Devoret, Journal of Superconductivity and Novel Magnetism **34**, 1633 (2021).
[13] B. Wielinga and G. J. Milburn, Phys. Rev. A **48**, 2494 (1993), URL `https://link.aps.org/doi/10.1103/PhysRevA.48.2494`.
[14] S. Puri, S. Boutin, and A. Blais, npj Quantum Information **3**, 1 (2017).
[15] P. T. Cochrane, G. J. Milburn, and W. J. Munro, Physical Review A **59**, 2631 (1999).
[16] T. L. Curtright, D. B. Fairlie, and C. K. Zachos, A concise treatise on quantum mechanics in phase space (World Scientific Publishing Company, 2013).
[17] H. J. Groenewold, in On the principles of elementary quantum mechanics (Springer, 1946), pp. 1–56.
[18] J. E. Moyal, in Mathematical Proceedings of the Cambridge Philosophical Society (Cambridge University Press, 1949), vol. 45, pp. 99–124.
[19] M. Hillery, R. F. O'Connell, M. O. Scully, and E. P. Wigner, Physics reports **106**, 121 (1984).
[20] W. B. Case, American Journal of Physics **76**, 937 (2008).
[21] M. C. Gutzwiller, Chaos in classical and quantum mechanics, vol. 1 (Springer Science & Business Media, 2013).
[22] L. D. Landau and E. Lifshitz, Course of theoretical physics, quantum mechanics non-relativistic theory (Pergamon, 1991).
[23] D. J. Griffiths and D. F. Schroeter, Introduction to quantum mechanics (Cambridge university press, 2018).
[24] M. Marthaler and M. Dykman, Physical Review A **73**, 042108 (2006).
[25] Y. Zhang and M. I. Dykman, Phys. Rev. A **95**, 053841 (2017).
[26] K. E. Cahill and R. J. Glauber, Phys. Rev. **177**, 1857 (1969), URL `https://link.aps.org/doi/10.1103/PhysRev.177.1857`.
[27] F. Iachello, in Neutron Capture Gamma-Ray Spectroscopy (Springer, 1979), pp. 23–35.
[28] F. Iachello, in Lie Algebras and Applications (Springer, 2015), pp. 53–56.
[29] H. J. Carmichael, Statistical methods in quantum optics 1: master equations and Fokker-Planck equations, vol. 1 (Springer Science & Business Media, 1999).
[30] H. J. Carmichael, Statistical methods in quantum optics 2: Non-classical fields (Springer Science & Business Media, 2009).
[31] H.-P. Breuer, F. Petruccione, et al., The theory of open quantum systems (Oxford University Press on Demand, 2002).
[32] J. Venkatraman, X. Xiao, R. G. Cortiñas, and M. H. Devoret, arXiv preprint arXiv:2209.11193 (2022).
[33] M. I. Dykman, C. M. Maloney, V. N. Smelyanskiy, and M. Silverstein, Phys. Rev. E **57**, 5202 (1998), URL `https://link.aps.org/doi/10.1103/PhysRevE.57.5202`.
[34] J.-M. Raimond and S. Haroche, Oxford University Press **82**, 86 (2006).
[35] R. R. Puri et al., Mathematical methods of quantum optics, vol. 79 (Springer, 2001).
[36] X. Xiao, J. Venkatraman, R. G. Cortiñas, S. Chowdhury, and M. H. Devoret, arXiv preprint arXiv:2304.13656 (2023).
[37] E. Wigner, Phys. Rev. **40**, 749 (1932).
[38] H. Weyl, The Theory of Groups and Quantum Mechanics (E. P. Dutton & Co., 1931).
[39] C. Zachos, Journal of Mathematical Physics **41**, 5129 (2000).
[40] N. H. McCoy, Proceedings of the National Academy of Sciences **18**, 674 (1932).
[41] J. Venkatraman, X. Xiao, R. G. Cortiñas, A. Eickbusch, and M. H. Devoret, Physical Review Letters **129**, 100601 (2022).
[42] M. Dykman, Fluctuating nonlinear oscillators: from nanomechanics to quantum superconducting circuits (Oxford University Press, 2012).
